# Cognitive Control Reflects Context Monitoring, Not Motoric Stopping, in Response Inhibition

**DOI:** 10.1371/journal.pone.0031546

**Published:** 2012-02-27

**Authors:** Christopher H. Chatham, Eric D. Claus, Albert Kim, Tim Curran, Marie T. Banich, Yuko Munakata

**Affiliations:** 1 Department of Cognitive, Linguistic and Psychological Sciences, Brown University, Providence, Rhode Island, United States of America; 2 The Mind Research Network, Albuquerque, New Mexico, United States of America; 3 Department of Psychology and Neuroscience, University of Colorado, Boulder, Colorado, United States of America; 4 Institute of Cognitive Science, University of Colorado, Boulder, Colorado, United States of America; University College London, United Kingdom

## Abstract

The inhibition of unwanted behaviors is considered an effortful and controlled ability. However, inhibition also requires the detection of contexts indicating that old behaviors may be inappropriate – in other words, inhibition requires the ability to monitor context in the service of goals, which we refer to as context-monitoring. Using behavioral, neuroimaging, electrophysiological and computational approaches, we tested whether motoric stopping per se is the cognitively-controlled process supporting response inhibition, or whether context-monitoring may fill this role. Our results demonstrate that inhibition does not require control mechanisms beyond those involved in context-monitoring, and that such control mechanisms are the same regardless of stopping demands. These results challenge dominant accounts of inhibitory control, which posit that motoric stopping is the cognitively-controlled process of response inhibition, and clarify emerging debates on the frontal substrates of response inhibition by replacing the centrality of controlled mechanisms for motoric stopping with context-monitoring.

## Introduction

Inhibition is critical for enabling controlled behavior: bad habits, unfamiliar situations, and dangerous environments often require that default behaviors be stopped and more context-appropriate actions performed [1]. Inhibitory control has been statistically equated with the behavioral and genetic variance common across multiple tests of cognitive and behavioral control [2]–[3]. Moreover, a particular domain of inhibitory control – response inhibition – has been exempted from the skepticisms surrounding other domains of inhibition [4], and specifically linked to the functioning of a particular frontal region (the right ventrolateral prefrontal cortex; rVLPFC). In this way, the study of response inhibition has supported theorizing that similar mechanisms may enable the inhibition of thoughts and emotions. Thus, modern theorizing is largely consistent with a hypothesis proposed 130 years ago: that “the centers of inhibition being thus the essential factor of attention, constitute the organic basis of all the higher intellectual faculties” [5].

However, effective inhibitory control not only requires actually stopping unwanted actions, thoughts, or emotions – it also requires the efficient detection of those contexts that indicate the need for these forms of stopping. To use an example from the domain of response inhibition, one's goal may be to cross a street; this requires actually crossing the street, and stopping these motor actions if oncoming traffic is approaching, but to do so the environment must be monitored so that this motoric stopping can be performed as appropriate. In other words, the environmental context must be monitored to support behavior that may be contingent on that context. Both motoric stopping and context-monitoring are also intermingled in the most precise laboratory assessment of response inhibition, in which subjects must cancel a prepotent or planned response after the presentation of a signal to stop [6]–[12]. The time that subjects require to stop an action, or “Stop Signal Reaction Time” (SSRT) can be estimated based on a formal model of the “stopping process,” although the model's estimate of this process intermingles the time spent detecting or interpreting the signal to stop with the latency for motoric stopping *per* se to take place [7]. Indeed, this process impurity is both widely acknowledged [e.g., 2] and implicit in the original proposal [7]. Thus, even within this well-studied domain of response inhibition, the so-called “stopping process” could largely reflect a controlled context-monitoring process that supports the detection and interpretation of the behaviorally-relevant signal to stop [8]–[11].

**Figure 1:**
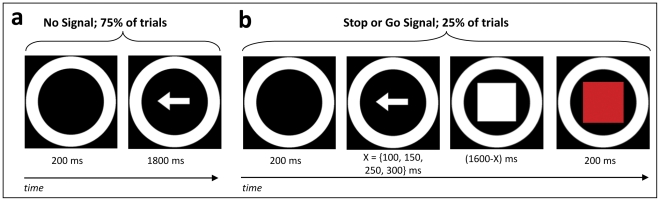
An illustration of the task design. Identical stimuli and trial structure were used across tasks in three separate experiments. In both the Stop and the Double Go tasks, most trials are “No Signal” trials where only a 2AFC decision is required (A). However, the tasks differ on “Signal” trials (B) where an additional stimulus, a white box, is presented with a variable inter-stimulus interval following the onset of the 2AFC stimulus. On Double Go_Signal_ trials, this additional stimulus indicates that the appropriate 2AFC button press be repeated. On Stop_Signal_ trials, this stimulus indicates that the 2AFC button press must be stopped. Thus, although only the Stop Task requires motoric stopping, both tasks share demands on context-monitoring.

To determine whether context-monitoring or stopping may constitute the cognitively-controlled process of inhibitory control, we focus on response inhibition as an example domain. We experimentally dissociate the motoric stopping that occurs during response inhibition from the context-monitoring processes that are also involved, by examining two tasks with identical context-monitoring demands, one of which requires motoric stopping and one of which does not. In both tasks, 75% of trials (“No Signal” trials) require a 2-alternative forced choice (2AFC; Fig. 1A); in the remaining 25% of trials (“Signal” trials), the 2AFC is followed by a behaviorally-relevant stimulus (the “signal”) after a variable delay (Fig. 1B). In the Stop Task, Signal trials require the stopping of motor responses on that trial. In contrast, in the “Double Go Task,” Signal trials require subjects to repeat their response for that trial as quickly as possible (see methods and Text S1). Thus, both tasks require monitoring for the context that signals what actions should be executed, but only the Stop Task explicitly requires motor actions to be stopped.

The cognitive control required for response inhibition is thought to rely on the prefrontal cortex, to be most crucial at the moment when motoric stopping is required, to be associated with substantial mental effort, to be recruited in a goal-directed fashion, and to support consistent individual differences. We assess each of these characteristics of cognitive control via behavioral, computational, hemodynamic, electrophysiological and pupillometric techniques to determine whether context-monitoring or motoric stopping may reflect the cognitively-controlled process recruited during response inhibition. Convergent evidence of this kind is necessary for making broad claims about the content of cognitive control because cognitive control cannot be unambiguously defined on the basis of any of these characteristics in isolation (e.g., neither prefrontal recruitment nor mental effort alone are sufficient). In addition, this convergent evidence allows us to make multiple points of contact with prior uses of these techniques in the domain of response inhibition, as outlined below.

We used functional magnetic resonance imaging (fMRI) to assess the recruitment of the prefrontal cortex in our tasks. Numerous previous fMRI studies have demonstrated transient activation within the right ventrolateral prefrontal cortex (rVLPFC) and the adjoining anterior insula during trials that require motoric stopping [1]–[2], [6], [8]–[12]. Collectively, this and related evidence has been interpreted to indicate that the rVLPFC is a dedicated substrate for inhibition, and that this function may also be deployed proactively to support behaviors like “responding with restraint” [12]–[13]. Alternatively, it is possible that these hemodynamic patterns reflect the context monitoring demands of the Stop task, which could also be deployed proactively as well as transiently at the moment a goal-relevant feature of the environment (e.g., a Stop Signal) is encountered. Recent work has begun to examine this alternative possibility using fMRI but has unfortunately yielded inconsistent results: either more [8], [11], less [7], [10] or roughly equivalent [9] rVLPFC activity is observed during the Stop task, in either overlapping [8], [11] or distinct [6], [10] subregions of the rVLPFC. In addition, none of these studies have examined whether the sustained component to the rVLPFC hemodynamic response could reflect a tonic and proactive process of context-monitoring (in which case sustained activity should also be observed in a context-monitoring task) rather than a process of responding with restraint. Finally, all of these studies have examined only the univariate patterns in hemodynamics, and have not assessed whether rVLPFC demonstrates multivariate commonality across tasks involving context-monitoring (as would be predicted by context-monitoring accounts), or whether any such commonality is relatively decreased on trials requiring motoric stopping (as would be predicted by stopping accounts). Below, we measured each of these aspects of the recruitment of the rVLPFC during the Stop and Double Go tasks to test these differing predictions of the context-monitoring and motoric stopping accounts.

We also assessed whether the event-related potentials commonly associated with response inhibition tasks, and often presumed to reflect motoric stopping processes, might instead reflect context monitoring processes. The most characteristic ERP from response inhibition tasks is the “Stop P3” or “No/Go P3,” a frontocentral positivity elicited following the onset of stimuli which demand motoric stopping [14]. We tested whether this “Stop P3” would be more strongly expressed during the Stop task than the Double Go task (as motoric stopping accounts would predict), and whether the correlation of all ERPs across these tasks would be reduced following the onset of the Signals (as would also be predicted by motoric stopping accounts). In contrast, accounts positing the centrality of context-monitoring to the Stop task would predict roughly equivalent frontocentral ERPs across these tasks, despite their differing demands on motoric stopping.

Finally, we assessed the task-evoked pupillometric response (TEPR), a well-validated measure of mental effort [15]–[16], to determine whether the relatively more effortful component to the Stop task reflects motoric stopping (in which case pupil diameter should be increased on Stop_Signal_ trials) or whether it might reflect the act of monitoring context for goal-relevant signals (in which case, pupil diameter may show a more complex pattern, such as a modulation of pupil diameter by the relevance of a monitored signal to the planned response). Previous work examining pupil diameter in the Stop task has utilized it mainly as a control measure of arousal in TMS studies [17]–[18].

To foreshadow our results, our results uniformly suggest that, during response inhibition, cognitive control is primarily engaged for the purpose of monitoring the environmental context in the service of goals, rather than for motoric stopping *per se*.

## Results

### Univariate fMRI Results

**Figure 2:**
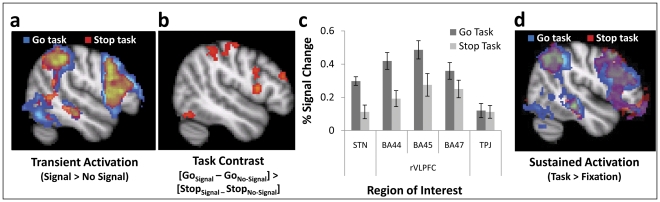
The Stop and Go tasks recruit overlapping neural substrates as revealed in both transient and sustained hemodynamics. Hybrid fMRI analyses revealed overlapping neural activity in the Stop and (Double) Go Tasks (A), with significantly more rVLPFC activity in the Go Task (B). ROI analyses for the contrast of Signal vs. No-Signal trials (C) revealed increased activity in the Go Task throughout a putatively stopping-specific network; this pattern did not generalize to regions with more general attentional functions (e.g., TPJ). Sustained rVLPFC activity was also observed across all trials within each task (D).

First, we found that context-monitoring rather than stopping explained the transient prefrontal contribution to response inhibition. Accounts which posit that motoric stopping is the controlled process during response inhibition tasks predict rVLPFC activation only in the Stop task, but event-related fMRI revealed that the Stop and Double Go tasks activated completely overlapping regions of prefrontal cortex (Fig. 2A), consistent with the tasks' shared context-monitoring demands. Specific regions of interest (ROIs) in the rVLPFC and interconnected subthalamic nucleus (STN) that have been proposed to be specific to the motoric stopping demands were uniformly more strongly recruited on Signal trials in the Double Go Task (Fig. 2B&C; STN: t(17) = 5.49, p<.0001; BA44: t(17) = 5.08, p<.0001; BA45: t(17) = 2.83, p = .012; BA47: t(17) = 2.5, p = .023), challenging any characterization of these areas as specialized for motoric stopping. A significantly different pattern was observed in areas thought to have a more general attentional role (e.g., the temporo-parietal junction; TPJ [19]–[20]; F(1,17) = 31.57, p<.0001), such that both tasks recruited this area equivalently. This equal recruitment of the TPJ across tasks indicates that decreased recruitment of the rVLPFC in the Stop task cannot be explained by globally-decreased activation during that task (e.g., as might result from fatigue; see also discussion in Text S1). Moreover, the increased recruitment of rVLPFC during the Double Go task is consistent with several recent findings, which also demonstrate that tasks involving both context-monitoring and response commission are associated with increased rVLPFC activity relative to tasks involving both context-monitoring and a demand to stop motor actions [7]–[10] (but see [6] and discussion, below).

Our hybrid fMRI design also allowed us to assess the extent to which neural regions were recruited in a sustained fashion across all trials within the Stop and Double Go tasks. Such sustained activity is potentially a hallmark of proactive context-monitoring processes. Indeed, this analysis revealed sustained hemodynamics in the rVLPFC during both tasks at the timescale of seconds-to-minutes (Fig. 2D), consistent with their shared sustained context-monitoring demands. In contrast, accounts positing that motoric stopping is the cognitively-controlled process during response inhibition predict no sustained rVLPFC activity in the Double Go task, since only response commission is required by that task, and “responding with restraint” is unnecessary.

### Multivariate Pattern Analysis

**Figure 3:**
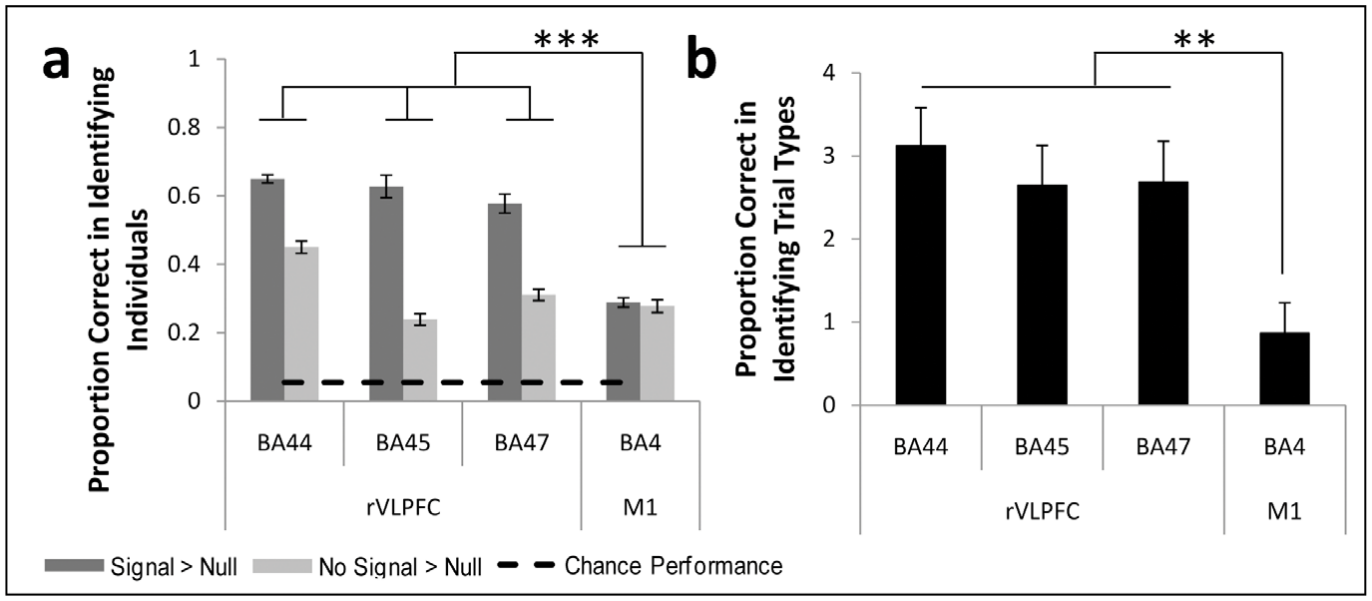
Multivariate pattern analysis reveals similar representations in the rVLPFC despite differing inhibitory demands. (A) rVLPFC was recruited in subject-specific but consistent ways regardless of stopping demands: individual differences in (Double) Go task hemodynamic activity also differentiated subjects in the Stop task. (B). rVLPFC showed trial-type-specific recruitment that was consistent across tasks, contradicting stopping-specific accounts of rVLPFC function. ** p<.0001 *** p<.005.

We next leveraged multi-voxel pattern analysis to determine whether the same information was encoded by rVLPFC regardless of whether motoric stopping is required by a given task. First, we trained classifiers to identify hemodynamic patterns that reliably predicted subject-specific patterns of rVLPFC activation in the Double Go task over 10 independent runs of the classifier (see methods in Text S1 and Figures S3 & S4). Classifiers readily generalized their training on the Double Go task to distinguish individuals in the Stop task, indicating that the rVLPFC is recruited in an individual-specific but consistent way across tasks. These patterns were significantly more consistent across tasks on Signal trials in the rVLPFC – precisely when and where context-monitoring processes are most crucial, but also when motoric stopping demands differ most across these tasks (Fig. 3A; BA44: t(9) = 13.5, p<.0001; BA45: t(9) = 11.39, p<.0001; BA47: t(9) = 12.35, p<.001). Critically, the increased cross-task similarity of Signal trials relative to No Signal trials was not observed in an area known to encode responses – primary motor cortex – and this pattern was significantly different from that observed in rVLPFC (F(1,9) = 85.12, p<.0001).

Although these results do not conclusively demonstrate that the cognitive processes engaged by both tasks are the same, they do demonstrate that the multivariate representations in the rVLPFC fail to show differential sensitivity to the explicit stopping demands imposed by Signal trials within the Stop task (in contrast to the multivariate patterns within primary motor cortex). This pattern contradicts the idea that representations in rVLPFC are specialized for the motoric stopping that is required on Signal trials in the Stop task (but not in the Double Go task). Thus, while No Signal trials are indeed similar across tasks (as revealed by the uniformly above-chance classification of individuals on these trials; light gray bars of Fig. 3A), Signal trials reveal processes that are particularly stable within individuals across these tasks, despite their different demands on motoric stopping, within the rVLPFC. This pattern is wholly consistent with the idea that similar context-monitoring processes are elicited by Signal trials within both tasks.

In a second multi-voxel pattern analysis, subject-specific classifiers were trained to decode the multivariate patterns that differentiate Double Go_Signal_ and Double Go_No-Signal_ trials. Classifiers generalized this training on the Double Go task to correctly identify Stop_Signal_ trials with 92–97% accuracy in the rVLPFC, significantly higher than the 59% accuracy achieved in primary motor cortex (F(1,17) = 9.413, p = .007). To control for the possible effects of classifier bias on this result, we utilized signal detection theory. Classifiers readily discriminated between Stop_Signal_ and Stop_No-Signal_ trials in the rVLPFC but not primary motor cortex in terms of d' (Fig. 3B; F(1,17) = 13.14, p<.005). Thus, while the Double Go classifier cannot be applied successfully to the Stop task in primary motor cortex (as one would expect given the tasks' different motor demands), it can be in the rVLPFC – indicating that the rVLPFC-mediated control process is similar across these tasks despite their different demands on motoric stopping.

### Event-related potentials

**Figure 4:**
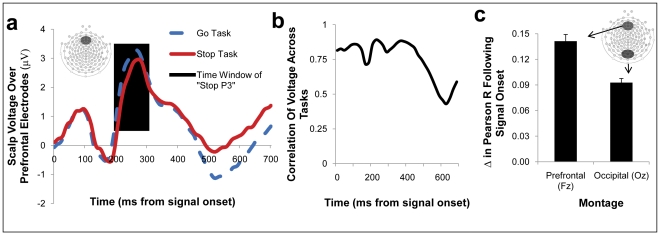
Prefrontal event-related potentials do not strongly distinguish the tasks. A prefrontal positivity peaking around 300 ms, known as the “Stop P3,” has been previously associated with stopping, but this component (darkened region of A) was significantly enhanced in the (Double) Go task. Individual differences in voltage were also highly correlated across tasks, indicating substantial overlap in the underlying cortical processes (B). Moreover, prefrontal correlations between the scalp voltage recorded across tasks were disproportionately increased following the presentation of the signal, relative to the increase in occipital correlations observed at the same time (C). This difference indicates increased cross-task similarity in prefrontal processing specifically at signal onset.

The ERPs evoked by our tasks also reflected context-monitoring demands rather than stopping demands. In particular, motoric stopping accounts predict that a prefrontal ERP called the “Stop P3” reflects stopping-specific processes [14] and should therefore be enhanced in the Stop task. However, the so-called Stop P3 was enhanced in the Double Go task, in direct contradiction to the stopping account (Fig. 4A; t(35) = 2.92, p<.03; see also Figures S5 and S6), but consistent with our observations of increased transient hemodynamics in the Double Go task relative to the Stop task (see Univariate fMRI results, above).

These ERPs from the Stop and Double Go tasks were not two distinct potentials masquerading as the same; individual differences in this ERP were also highly correlated between tasks (Fig. 4B). Critically, correlations between the ERPs elicited by each task were disproportionately increased over prefrontal electrodes, relative to occipital electrodes, following signal onset, when context-monitoring is most required but stopping demands differ most (Fig. 4C; F(1,98) = 12.59, p = .001).

### Pupillometry

**Figure 5:**
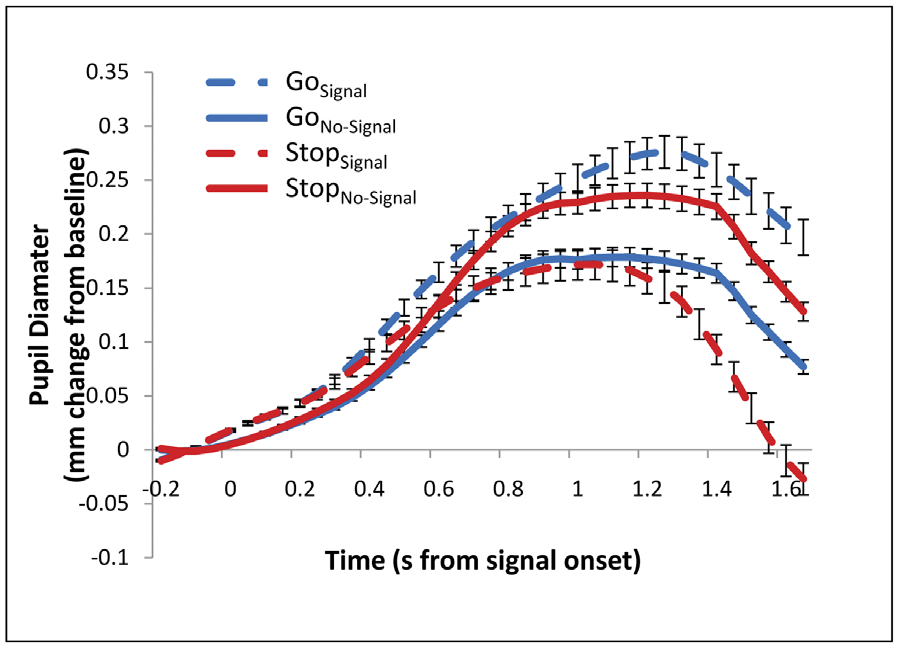
Patterns of mental effort assessed via pupillometry indicate that effort matches demands on context-monitoring, not stopping, and is modulated by the relevance of the infrequent stimulus to the planned response. In particular, stopping a response (Stop_Signal_ trials) was associated with more mental effort was required by monitoring for the appearance of stimuli that would demand stopping (Stop_No-Signal_ trials) than by stopping itself (Stop_Signal_ trials) or by monitoring for the appearance of stimuli that would demand an additional act of going (Go_No-Signal_ trials).

We also found that context-monitoring, not motoric stopping, explains the patterns of mental effort elicited during our tasks. We measured pupil diameter, a psychophysiological index of mental effort [15]–[16], following the onset of a signal (or the average signal onset time in the case of No-Signal trials; Fig. 5). Averaging across all time points, mental effort was less for stopping than for monitoring for signals that fail to appear (Stop_Signal_<Stop_No-Signal_t(85) = 7.00, p<.001; Stop_Signal_<Double Go_No-Signal_ t(85) = 2.07, p<.05). Mental effort was also less for context monitoring and motoric stopping than for context monitoring and an additional act of going (Stop_Signal_<Double Go_Signal_ t(85) = 13.67, p<.001). Finally, mental effort was greater when monitoring for signals that would require a change to the planned response than when monitoring for those that would not (Stop_No-Signal_ >Double Go_No-Signal_ t(85) = 10.25, p<.001), a result which also rules out global reductions in effort during the Stop task (e.g. from fatigue; see also Text S1). Thus, motoric stopping is not itself associated with effort beyond that required for the processes involved in other trial types, contrary to the idea that motoric stopping of a response constitutes a particularly effortful component of response inhibition. Instead, context-monitoring demands are more central to mental effort, and this relationship is modulated by the relevance of the monitored stimulus to the planned response.

### Model-based decomposition of behavior and correlations with brain activity

**Figure 6:**
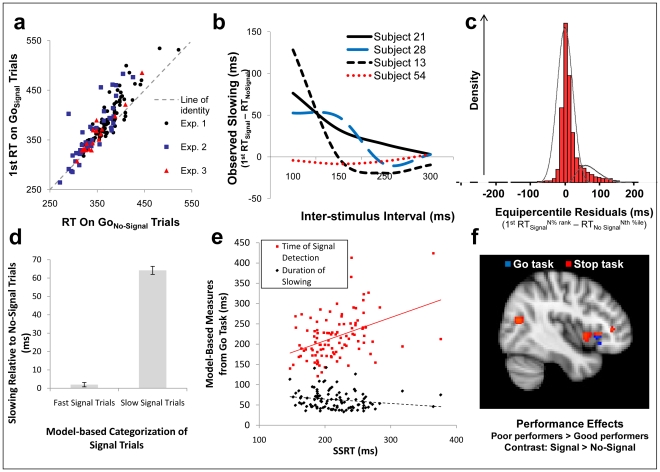
Mixture model analyses separate slowed from unslowed trials in the Go task, and demonstrate this slowing is not the source of the commonality across tasks. Response slowing was observed in the Double Go task (A), perhaps suggesting that stopping is not associated with differential mental effort or prefrontal activity because it is an automatic consequence of detecting an infrequent stimulus. Critically, this slowing was dependent on ISI; indeed, large individual differences were observed in the shortest ISI to yield zero slowing (B contains data from four representative subjects). A subtraction of reaction times on (Double) Go_No-Signal_ trials from those with a corresponding percent rank on (Double) Go_Signal_ trials reveals a pronounced positive skew to these equipercentile residuals (C), indicating that some proportion of reaction times on Go_Signal_ trials are disproportionately delayed. Trials undergoing this slowing were identified as those more likely to come from a distribution not centered on zero, as determined through a two-component mixture model (see overlaid lines on histogram in C). This procedure adequately separated the slowed and unslowed distributions, as revealed by zero significant difference between Go_Signal_ trials categorized as unslowed and their corresponding reaction times in the Go_No-Signal_ distribution, but a large difference between Go_Signal_ trials categorized as slowed and their corresponding reaction times in the Go_No-Signal_ distribution (D). From this we estimated two individual differences: how long subjects are slowed (duration of slowing; DoS) and the time at which signals are detected (time of signal detection; TOSD). Only TOSD positively correlated with SSRT, whereas DoS showed a slight negative correlation, indicating that the slowing experienced by subjects in the Double Go task cannot be the source of shared variance between the Stop and Double Go tasks (E). Brain-behavior correlations confirmed this conclusion: SSRT and TOSD, but not DoS, overlapped in their correlations with neural activity only in the rVLPFC (F).

Stopping is not associated with differential mental effort or prefrontal recruitment, contrary to what might be predicted from stopping-centric accounts of cognitive control. This pattern of results could imply that motoric stopping is not a cognitively-controlled process, given the widely-held assumption that controlled, goal-directed processes recruit the prefrontal cortex and require differential mental effort. Consistent with this idea, subjects appear to engage in reflexive stopping even on the Double Go task, where such stopping runs contrary to instructed goals. Specifically, although Double Go_Signal_ trials require that subjects commit a subset of the motor responses required on Double Go_No-Signal_ trials, subjects were nonetheless slower to provide even their first response to stimuli when they were followed by the signal than when they appeared alone (Double Go_Signal_^1st RT^>Double Go_NoSignal_^Only RT^; t(148) = 9.59, p<.0005; Fig. 6A). To the extent that this behavioral slowing in the Double Go task reflects some transient stopping, it runs contrary to subjects' goals in the Double Go task and therefore might not be engaged in a controlled or goal-directed manner.

On the other hand, the presence of goal-inconsistent slowing during the Double Go task does not by itself refute the idea that motoric stopping can be a controlled process in this task or in others. Indeed, one alternative interpretation of this slowing is that it does in fact reflect a controlled and goal-directed process: it may be an attempt to stop or replace the motor plan required on Double Go_No-Signal_ trials (i.e. the motor plan for “respond once” is stopped or replaced with the motor plan for “respond twice”). We assessed this possibility with a model-based decomposition of subjects' behavior; however, the results of this analysis argue against this possibility, and further show that the efficiency of subjects' context-monitoring, rather than the efficiency of motoric stopping or motor plan replacement, shares a closer relationship with SSRT.

To assess the alternative accounts, we developed a formal model of context-monitoring and stopping by building on the classic race model of the Stop task [7] (see also Text S1 and Figure S7A) in order to precisely estimate the duration of motoric slowing experienced by subjects in the Double Go task, as well as exactly which trials underwent such slowing (Figure S7B). The race model of the Stop task posits that responses undergo inhibition when a stopping process, triggered by the onset of the Stop signal, completes *before* the “going” processes triggered by the onset of the 2AFC stimulus. The race between stopping and going processes is the model's namesake, and is supported by the monotonically-decreasing relationship of interstimulus interval (ISI) to successful inhibition: larger ISIs give the “going” process an increasing advantage in the race, and thus leads to less successful inhibition. We observed a similar phenomenon in our Double Go task, such that increasing ISIs led to less slowing of first responses; this effect was visible at the group level (Figure S7C) but also even at the level of individual subjects (Fig. 6B), who showed substantial variability in the earliest ISI to yield zero observable slowing.

We utilized this behavioral variability to estimate individual differences in Double Go task performance. First, we estimated the probability that each trial belonged to either the “slowed” or “unslowed” distributions of reaction times. This categorization was accomplished by fitting a mixture model to the difference between reaction times of Double Go_Signal_^1st RT^ and Double Go_NoSignal_^Only RT^ trials of corresponding percent rank. To the extent these reaction times come from the same (i.e., unslowed) distribution, these equipercentile residuals should be centered on zero; however, there was pronounced positive skew (Fig. 6C), indicating that a substantial proportion of trials did undergo slowing. We considered as “slowed” those trials that were marginally less likely to come from a Gaussian distribution centered on zero, relative to an alternative distribution with a positive mean (see overlaid curves on Fig. 6C, and Text S1). This method clearly separated “slowed” from “unslowed” trials on the basis of the first RT on Double Go_Signal_ trials: “unslowed” trials showed approximately zero slowing relative to corresponding trials within the No Signal distribution, whereas “slowed” trials were significantly longer than corresponding trials within the No Signal distribution (Fig. 6D).

Next, we estimated for each subject the amount of time that must elapse after signal presentation until responses are categorized as “slowed” (yielding the time of signal detection [TOSD], our measure of context-monitoring), and the difference between that subjects' “slowed” and “unslowed” reaction times (yielding the duration of slowing [DoS], our measure of stopping from the Double Go task). If motoric stopping (or, equivalently, motor plan replacement) is controlled, and initiated in this controlled fashion in the Double Go task, then the process of motoric stopping or motor plan replacement should should cease (as estimated by DoS, in the Double Go task) in proportion to how quickly competing motor plans can be stopped, as assessed by SSRT in the Stop task. That is, the “controlled motoric stopping” and “controlled motor plan replacement” accounts both predict that DoS and SSRT should be positively correlated.

However, DoS and SSRT were not positively correlated – they instead showed a weak negative correlation (Pearson R = −.188, p = .048; Fig. 6E), in direct contradiction to the prediction motivated by these alternative accounts. SSRT was instead positively correlated only with TOSD – i.e., the efficiency with which signals could be detected (Fig. 6E; R = .418, p<.0005) – as predicted by accounts which posit that context-monitoring underlies the commonalities of the Double Go and Stop Signal tasks. This positive relationship persisted when controlling for DoS (R = .410, p<.0005), indicating that the overlapping variance in TOSD and SSRT does not reflect motoric stopping or motor plan replacement. Strikingly, this relationship of context-monitoring to SSRT was also regionally-specific: SSRT and TOSD overlapped in their relationship to hemodynamics only within the rVLPFC (Fig. 6F).

A second, independent assessment of the origin of the observed commonalities across our tasks is also enabled by our formal model. Specifically, the model identifies exactly which trials undergo motoric stopping/slowing within the Double Go task, and thus permits these trials to be excluded from analysis. To the extent that similar hemodynamic, electroencephalographic, and pupillometric patterns are observed when these “slowed” trials are excluded, it would suggest that the commonalities across our tasks do not reflect a motoric stopping process common to these tasks.

Consistent with the claim that a common and cognitively-controlled process of context-monitoring – and not a common process of motoric stopping – underlies the commonalities of our tasks, a complete re-analysis of the data without such “slowed” trials replicated all of our primary results: the increased transient hemodynamic response in the rVLPFC during the Double Go task, the prominent sustained hemodynamic activity observed in that task, the multivariate hemodynamic commonalities across tasks, the increased Stop P3 response in the Double Go task, the strong correlations of scalp voltage across tasks as well as the selective increase in those correlations over frontal electrodes following signal onset, and yields qualitatively similar patterns of mental effort (see Text S1 and Table S4). This analysis further substantiates our conclusion that context-monitoring, not motoric stopping, reflects the cognitively-controlled component of this canonical response inhibition task.

## Discussion

By matching our tasks on all characteristics except motoric stopping demands [6], [8]–[12], [19]–[21], we find that monitoring context for behaviorally-relevant signals, not stopping, is the more effortful, controlled, and prefrontally-based process engaged during a canonical test of response inhibition. All of these results replicated even when utilizing only the trials that were categorized as “unslowed” from the Double Go task, indicating that the slowing in that task was not the origin of the hemodynamic, electroencephalographic, and pupillometric commonalities. This conclusion is consistent with recent evidence that the behavioral slowing expressed in context-monitoring tasks is not related to hemodynamics in rVLPFC, nor to that in any portion of lateral prefrontal cortex [8]. In contrast, SSRT was instead more closely related to our measure of context monitoring, TOSD. Although these two measures are calculated in mathematically analogous ways, these calculations are nonetheless performed on tasks with quite dissimilar demands on inhibitory control. This correlation is surprising given that response inhibition tasks can fail to correlate even with superficially-varying versions of themselves [22].

It nonetheless remains possible that the prefrontal cortex could subserve some form of motoric stopping, or motor plan replacement, or that motoric stopping could in other contexts or tasks be cognitively controlled. Our results indicate only that there is no need to assume that motoric stopping occurs in a cognitively controlled fashion within the canonical task of response inhibition, the Stop task. Instead, many of the phenomena from this task – including both transient and sustained hemodynamics, multivariate patterns in those hemodynamics, event-related potentials, mental effort as quantified through pupillometry, and the primary behavioral measure from this task – seem to primarily reflect this task's demands on context monitoring processes. More broadly, our conclusions are also in line with those drawn on the basis of comparisons of the same Double Go task we used above [11] and alternative context monitoring tasks [8]–[10], [23]–[24] with the Stop Signal task, as described below.

### Relation to Recent Work

Our study addresses the evolving debate on the functional specialization of the rVLPFC [6], [8], [10], [18]–[21], [25] in three ways: by developing a formal model, by distinguishing subprocesses within these tasks that may have led to otherwise unresolved discrepancies across previous findings, and by testing a question of different scope: whether by any major criteria, motoric stopping could be considered a specific cognitive control mechanism utilized during response inhibition. Previous formal models did not separately account for motoric stopping and monitoring, or even explicitly distinguish between them [7]. Previous empirical attempts to dissociate monitoring and motoric stopping yielded conflicting results: less, more, or equivalent recruitment of either the same or separable subregions of rVLPFC [8]–[11]. Finally, previous neuroimaging work has focused largely on transient prefrontal hemodynamics [8]–[9], [11], [19]–[21] (but see [10] for an exception). By investigating not only transient but also the sustained and effortful components to inhibitory control, their goal-directedness, and the extent to which they drive individual differences in behavior, event-related potentials, and multivariate hemodynamics, we demonstrate the importance of context-monitoring as a mechanism of cognitive control.

Nonetheless, our results may seem to stand in contrast to some conflicting findings of previous work. Below, we step through these findings and describe how the context-monitoring account may offer a consistent way to understand these otherwise contradictory results, with respect to issues of statistical efficiency in estimating the hemodynamic response, potential dissociations between inferior and superior rVLPFC, the role of arousal, and the potential importance of goal replacement in the Stop task.

**Statistical Efficiency.** In previous neuroimaging work that included two types of Signal trials within the Stop task – signal trials that require stopping and “distractor” signal trials which indicate no change to the planned response – the latter activated parts of the rVLPFC that are either spatially indistinguishable [8] or spatially distinct [6], [9] from the portions of the rVLPFC recruited by the former. These conflicting results may indicate that the hemodynamic responses to these two types of signal trials were estimated with differential efficiency.

Indeed, when statistical efficiency is precisely matched across tasks, as in one previous study [8], response inhibition in the Stop task is actually associated with a decreased transient hemodynamic response relative to tasks involving response commission (consistent with our ERP and fMRI results). This previous study also demonstrated that rVLPFC activity was increased to infrequent stimuli that required either response commission or response inhibition, relative to infrequent stimuli that required no overt behavior [8], [11]. This result can be viewed as consistent with context-monitoring, which posits the rVLPFC is involved in the detection and interpretation of behaviorally-relevant stimuli to guide the selection of action.

**Dissociations between inferior/superior lateral PFC.** Some studies suggest that a region of superior rVLPFC (in particular, the right inferior frontal junction) may be more crucial for processes analogous to context-monitoring than an inferior section of rVLPFC, which is more crucial for stopping [6], [10], [18]. In our results, these inferior and superior rVLPFC regions were distinctly activated, but both foci were more strongly activated on Double Go_Signal_ trials than Stop_Signal_ trials (Fig. 2B). Thus, while inferior and superior rVLPFC could differentiate in principle, a simple monitoring vs. motoric stopping dichotomy is not sufficient for explaining the patterns observed here.

Instead, the apparent dissociations between superior and inferior rVLPFC observed previously may represent differences in efficiency across trial types, as described above, or the absence of a viable model to analyze behavior in paradigms with infrequent response commission trials. Specifically, the behavioral model used in one recent TMS study of the functional specialization of the rVLPFC [18] assumes that the first response is unaffected by the appearance of the signal. However, we found that the first response is slowed by the appearance of a signal (Fig. 6A), and that measures extracted from these dynamics correlate selectively with rVLPFC (Fig. 6F). These results challenge the assumptions of the behavioral model in the TMS study, and the associated claims of dissociations between inferior and superior rVLPFC [21].

A second recent TMS study applied a “conditioning” pulse to the rVLPFC prior to a “test” pulse to primary motor cortex, and demonstrated a reduction in the observed motor-evoked potential (MEP) [26]. The authors interpreted their results to reflect a direct inhibitory role of the rVLPFC on M1. However, similar effects have been observed with conditioning pulses to dorsal prefrontal cortex [27]. It is thus likely that these TMS effects reflect relatively general mechanisms (e.g., short- or long-interval intracortical inhibition) that are not functionally specific to the rVLPFC.

**The role of arousal.** Previous work has reported a null effect of TMS to rVLPFC on pupil diameter using a relatively small sample of 17 subjects [17], contrary to what might be expected if effortful monitoring processes were disrupted by rVLPFC TMS. However, we note that TMS did lead to a consistent reduction in pupil diameter on correctly inhibited trials across all time points (Fig. 6D of [17]). Moreover, subsequent work has identified a generalized arousal effect of TMS [18], which may have increased pupil diameter and thus masked decreases in pupil diameter after disruption of context-monitoring. Thus, to the extent any conclusions can be drawn on a null effect in a small sample, this previous result may suggest that the disruption of effortful context-monitoring processes (and consequently, decreases in pupil diameter) was only partially offset by the generalized arousal resulting from rVLPFC TMS.

**The relationship of response inhibition to goal-switching.** Some theoretical accounts of Stop task performance emphasize the importance of goal-switching, such that a “Go goal” must be replaced with a “Stop goal” on Stop_Signal_ trials [28]. However, factor analytic studies of individual differences in response inhibition (including the Stop task) and those in task-switching and working memory updating indicate that there is significant switching- and updating-specific variance in individual differences that is non-overlapping with that in performance on response inhibition tasks [3]. The prevailing interpretation of these findings is that performance on response inhibition tasks is primarily driven by cognitive control processes supported by active maintenance mechanisms, and are thus common across all executive function tasks, rather than being specific to goal- or task-switching, or to working memory updating processes [3]. Our ongoing neurocomputational modeling work indicates that such active maintenance mechanisms, used in the service of context-monitoring, may indeed be sufficient for explaining detailed patterns of performance on the Stop task. This conclusion is further consistent with the fact that task-switching and Stop task performance do not influence one another (i.e., their effects are additive [29], but see also [30]), a result which argues against the idea that a controlled goal-switching process is operative during the Stop task.

### Broader Implications

Our results contradict a long-standing account of cognitive control in response inhibition tasks [1]–[3], [5]–[6], [12]. Because the mechanisms supporting response inhibition are thought to underlie many forms of self-control, our results could be taken to imply that context-monitoring is also central to the cognitive demands of other inhibitory domains. However, future work should assess this implication directly by comparing context-monitoring processes with alternative forms of stopping (e.g., mnemonic stopping in the case of memory inhibition tasks).

Although the neural computations that support the constituent processes of response inhibition – including its motoric stopping and context-monitoring components – remain to be fully specified, our experiments do indicate that a role for the rVLPFC in transient stimulus processing [1]–[2], [6], [8]–[12], [18]–[21], [23]–[5] is not mutually-exclusive with the sustained prefrontal dynamics emphasized by neuropsychological and neurocomputational theories [31]–[33]. Relatedly, we cannot rule out the possibility that additional important processes could be involved in the Double Go task, such as goal-switching (though see discussion above) or other as-yet unspecified control operations. We claim only that such assumptions are unnecessary to explain our results, nor those from other recent demonstrations of similarities between the Stop task and closely matched (yet non-inhibitory) tasks that involve only single responses [8]–[9] or responses across multiple effectors [18].

Nonetheless, even at its current level of detail, the context-monitoring account of rVLPFC function does accord with numerous emerging taxonomies of prefrontal organization. According to one recent taxonomy [33], ventral areas of the prefrontal cortex are particularly important for contextual processing of stimulus significance (broadly speaking, “what” processing) whereas more dorsal areas may be particularly important for the processing contextually-appropriate responses to those stimuli (“how” processing”). The putative localization of context-monitoring to rVLPFC is fully compatible with this framework. To the extent that response-related “stopping” processes have a dedicated prefrontal substrate, this taxonomy predicts that such processes should localize to areas dorsal to the rVLPFC, some of which project to the STN with similar or greater density [34]. However, our results do not suggest that dorsal areas are differentially associated with stopping, consistent with the wider literature [2], [6]–[10], and further supporting our conclusion that motoric stopping does not have a dedicated lateral prefrontal substrate within the Stop task. Thus, while motoric stopping may instead be subserved by a collection of heterogenous mechanisms, including the subthalamic nucleus and primary motor cortex, the rVLPFC's interaction with these circuits appears to derive more from its role in “what” processing (and perhaps with associated temporoparietal connectivity), rather than from a role that is more integrated with response demands (whether inhibitory or not) [33].

This context-monitoring role of rVLPFC may also be understood as arising from the proximity of rVLPFC to the anterior insula, which appears to monitor interoceptive information [35], in some cases proactively [36]. The anterior insula also shows greater hemodynamic responses to demands on action selection than to demands on motoric stopping, and is thought to be tightly integrated with the rVLPFC [37]. Thus, a basic mechanism in anterior insula for monitoring the internal significance of upcoming stimuli may have been evolutionarily adapted for use in monitoring the goal-relevance of stimuli for action selection in the nearby rVLPFC. These representations may even be tightly integrated, such that context monitoring can be effectively recruited when it counts most – under conditions of threat or pain. Indeed, target detection is improved when the targets are predictive of pain, an effect that is associated with greater activity in both rVLPFC and anterior insula [37].

The context-monitoring account is also compatible with recent revisions to a classical taxonomy of the effects of prefrontal insult, in which the inhibitory deficits arising from right lateral prefrontal damage are now explained as monitoring deficits instead [31]. The match between our findings and those motivating this taxonomic revision may indicate the need to rethink a broad range of putative inhibitory deficits. For example, focal rVLPFC damage can lead to poor target detection, such that even when the location of an upcoming target is cued before trial onset, this location is not effectively monitored following the onset of any stimulus [38]. While this deficit might reflect problems with inhibiting locations in space, our results suggest this patient's focal rVLPFC damage may have yielded a deficit in monitoring contextually-appropriate locations in the service of target detection and action selection.

In addition to the significance of our result for understanding neural insult, our result may also impact the treatment of pathological impulse control deficits (e.g. as in substance abuse or attention-deficit hyperactivity disorder [ADHD]). Specifically, our result suggests that pathological impulse control deficits might not reflect a failure to stop in particular, but rather the more effortful and prefrontal processes involved in context-monitoring. For example, ADHD may be associated with a monitoring deficit in which many stimuli, regardless of their behavioral-relevance, are thought to warrant attention. This prediction is supported by the finding that ADHD is more strongly associated with increased reaction time variability, as might result from a context-monitoring deficit, than with deficits in tasks that require stopping [39]. Relatedly, the resistance of response inhibition to improvement via training [40] may reflect that monitoring context for contingent action selection, not the act of stopping, is the controlled process to be targeted for effective intervention.

## Materials and Methods

### Ethics Statement

All research was approved by the institutional review board at the University of Colorado, and written informed consent was obtained from all participants.

### Participants

For experiment 1, 86 subjects (mean age 19.11 years; SD = 1.17 years; 32 males) were recruited using the University of Colorado undergraduate research pool and successfully completed the Double Go and Stop Tasks. 2 subjects failed eyetracker calibration and were excluded from pupillometric analyses. For experiment 2, 45 subjects (mean age 19.86 years; SD = 2.21; 23 males) were recruited using the University of Colorado undergraduate research pool and successfully completed the Double Go and Stop Tasks. 7 of these subjects were excluded from ERP analysis for artifacts caused by excessive blinking (>60% of trials). For experiment 3, 19 subjects (mean age 23.3: SD = 4.4; 10 males) were recruited from the local community and successfully completed the Double Go and Stop Tasks. One subject was excluded from fMRI analyses due to motion artifact.

### Behavioral Task

All subjects in all experiments completed the Double Go Task prior to completing the Stop Task. This fixed task order was adopted for reasons described in Text S1 – in particular, the use of a fixed task order is ideal for the investigation of individual differences [3], which was a central goal of the study reported here. Nonetheless, appropriate precautions were taken to prevent the contamination of experimental effects with cognitive phenomena that might arise from the fixed task order (e.g., the use of within-task baselines are used for all pupillometry, ERPs, and fMRI analyses, so as to control for the relatively general effects of phenomena like fatigue).

In all respects the Double Go and Stop Task were identical within any given experiment (e.g., the precise interstimulus and intersignal intervals, the presence of “null” trials, etc), with the following exception: subjects are naturally aware of when they fail to successfully stop a response, but seem unaware of their relative speed on trials with the infrequent stimulus. To avoid any possible mismatch across the two tasks owing to this difference, we provided explicit feedback on all signal trials. Specifically, in the Double Go Task, the signal turned red if subjects were slower than their average running RT (experiments 2 & 3); in experiment 1 this was presented as sham feedback. (Double Go task trials with categorically incorrect responses – such as a failure to respond twice on Signal trials, or anything but a single correct response on No Signal trials – were extremely rare and excluded from all analysis). Similarly, in the Stop Task, the signal turned red if subjects failed to successfully stop their response on that trial (in all experiments). Additional cross-experiment differences in our tasks suggest the generality of our results across minor variations in experimental procedure (see Figure S1 & Table S1).

### Statistical Analysis of fMRI

Data were acquired with a 3T GE Signal whole-body MRI scanner at the University of Colorado Health Sciences Center, using T2-weighted echo-planar imaging (EPI) (TR = 2000 ms, TE = 32 ms, flip angle = 70°). Additional acquisition details are available in Text S1.

Image pre-processing and analyses were conducted with FSL (FMRIB's Software Library). The first six volumes of each run were discarded to allow the MR signal to reach steady state, the remaining images in each participant's time series were motion corrected using MCFLIRT, and non-brain voxels were removed using a brain extraction algorithm (BET). The data series was spatially smoothed with a 3D Gaussian kernel (FWHM = 5 mm), intensity normalized for all volumes, and high-pass filtered (s = 50 sec).

After statistical analysis of each time series (details of the regression model are available in Text S1), statistical maps were normalized into the MNI-152 stereotaxic space using FLIRT (FMRIB's Linear Image Registration Tool). Parameter estimates (PE) were transformed into a common stereotaxic space using the above-mentioned three-step registration prior to the group analyses with FLAME (FMRIB's Local Analysis of Mixed Effects). Z-statistic images were thresholded using clusters with z >2.58 as well as a whole-brain corrected cluster significance threshold of p<.05 using the theory of Gaussian Random Fields.

ROIs for Brodmann areas were anatomically defined using the Talairach labeled atlas (see Figure S2), and mean percent signal change was extracted using FSL's featquery tool. The subthalamic nucleus was anatomically defined using a 10 mm^3^ region centered on the MNI coordinates previously used in the Stop Task to interrogate BOLD in the STN (10,−15,−5) [41]. The TPJ was anatomically defined using a 30 mm^3^ region centered on the MNI coordinates (−54, −52, 30) previously observed in a target detection task [42].

Pattern classification analyses were conducted on the beta-weights resulting from the above fMRI analysis pipeline, with four minor exceptions. First, the BOLD data were not spatially smoothed; second, the PEs were not statistically thresholded; third; the PEs were z-transformed across all voxels within a given ROI for each subject, to ensure that the classifiers were forced to operate on the basis of distributed patterns of activation instead of overall magnitudes. Finally, voxels with z-values falling outside of +/− 4.5 were windsorized. Classifiers were implemented as neural networks in Emergent [43]; separate networks were then trained, using Hebbian and Contrastive Hebbian learning, for each ROI (and therefore differed in terms of the number of input units), and for identifying which individuals generated the data vs. what trial type the data was estimated from (and therefore differed in terms of the number of output units) but all other aspects of the network architecture were the same. See Text S1 for full details on classifier implementation.

**Statistical Analysis of ERPs.** During the Double Go and Stop Tasks scalp voltages were recorded with a 128-channel geodesic sensor net [44]. Amplified analog voltages (0.1- to 100.0-Hz bandpass) were digitized at 250 Hz. Individual sensors were adjusted until impedances were less than 50 k. The EEG was digitally low-pass filtered at 40 Hz. Trials were discarded from analyses if they contained incorrect responses, eye movements (eye channel amplitudes over 70 V), or more than 20% of channels were bad (average amplitude over 100 V or transit amplitude over 50 V). Individual bad channels were replaced on a trial-by-trial basis with a spherical spline algorithm. EEG was measured with respect to a vertex reference (Cz), but an average-reference transformation was used to minimize the effects of reference-site activity and accurately estimate the scalp topography of the measured electrical fields. The average reference was corrected for the polar average reference effect [45]. ERPs were obtained by stimulus-locked averaging of the EEG recorded in each condition. ERPs were baseline-corrected with respect to a 200-ms prestimulus recording interval. These baselines were calculated separately for each task, thereby controlling for nonspecific effects like fatigue.

Where montages are used, the occipital montage was centered on Oz (including Oz, O1, O2, and the contiguous set of electrodes 76, 70, 74 and 82) and the frontal montage was centered on Fz (including Fz and the contiguous set of electrodes 4, 5, 10, 12, 16 18 and 19). For scalp-wide voltage correlations (Fig. 4B) we calculated Pearson's R across tasks at every time point as the variance shared between the subjects x electrode matrix across tasks. Thus, this correlation reflects changes in voltage that covary across tasks in the same subjects at the same electrode sites. For montage-based voltage correlations (main text Fig. 4C) we calculated Pearson correlations separately for the frontal and occipital montages both before and after signal onset.

**Statistical Analysis of Pupillometry.** Pupil diameter was recorded continuously during the Double Go and Stop Tasks via a Tobii X50 infrared eyetracker calibrated to each subject. Sampling at 50 Hz was synchronized to fixation onset, and pupil diameter was calculated as the average diameter of successfully-tracked eyes for each sample. Baseline measurements of pupil diameter were calculated as the average diameter during the 200 ms preceding the onset of each signal (or the corresponding time period for no signal trials); this value was subtracted from the averaged samples recorded following the onset of the signal (or the average signal onset for no signal trials). Baseline periods were calculated independently for the Stop and Double Go tasks, providing a within-task baseline to control for nonspecific cognitive effects like fatigue. These normalized, averaged pupil diameter samples were then smoothed using a box-car filter with width of 60 ms.

**Statistical Analysis of Behavior – Double Go Task.** In the Double Go Task, all RTs falling below 150 ms or above 750 ms were excluded from analysis, as well as those on No Signal trials falling outside of 3.5 standard deviations of the iteratively-calculated mean for each subject. RTs were only analyzed on correct trials (i.e., trials in which two responses of the correct type were provided on Signal trials, and where one and only one response of the correct type was provided on No Signal trials).

Individual differences were extracted from the Double Go task using a mixture model-based adaptation of the classic race model of the Stop task (see also Text S1). Specifically, to classify individual trials as slowed or unslowed, we first decomposed the distribution of equipercentile residuals into two underlying distributions: a Gaussian distribution with a mean of zero (corresponding to unslowed first RTs), and a Gamma distribution (corresponding to the slowed first RTs). The two free parameters to the Gamma and the one free parameter to the Gaussian were fit in a fixed-effects analysis using maximum likelihood estimation via with the Nelder-Meade simplex algorithm [46]–[47]. The maximum likelihood fit is illustrated as overlaid lines on the residual histogram (Fig. 6C), which was relatively stable across multiple optimizations with different starting parameters and yielded a better overall fit (see Table S1) than a single Gaussian in terms of the Bayesian Information Criterion (BIC), calculated as:
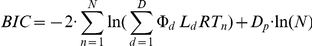
Where *N* is the total number of observations, *D* is the total number of distributions fit, D_p_ is the total number of free parameters used in fitting those distributions, 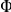 *_d_* is the weight of the *d* ^th^ distribution, and *L_d_*(*RT_n_*) is the likelihood of the *n* ^th^ RT given the best fit parameters for the *d* ^th^ distribution (μ and σ for Gaussian and *k* and Θ for Gamma).

We next categorized individual trials as slowed or unslowed using the likelihood of observing each RT under either of the two fitted distributions. RTs were categorized as slowed if there was even weak evidence in favor of the RT belonging to that distribution (as quantified by a difference in BIC of ≥2.35); otherwise RTs were categorized as unslowed. Other standards of evidence lead to similar results as those presented here, but do not as cleanly separate the slowed and unslowed trials (c.f. Fig. 6D).

To calculate TOSD, we subtracted the signal delay from the *n* ^th^ percentile of no signal trial RTs, where *n* corresponds to the proportion of RTs classified as unslowed at that signal delay. This approach is conceptually identical to that used to calculate SSRT in the race model, in which the signal delay is subtracted from the *n* ^th^ percentile of No Signal RTs, where *n* corresponds to the proportion of unsuccessful stop trials at that signal delay. TOSD was calculated for each subject as the median of these estimates across all signal delays. This estimate was unreasonably high for subjects for whom no RTs had been classified as slowed (n = 34 out of 150), so in those cases we used the minimum estimate of TOSD across all signal delays.

We then calculated the duration of slowing as the average difference between RTs classified as slowed and RTs of corresponding percent rank in the no signal RT distribution; subjects for whom no RTs had been classified as slowed were excluded from all analyses involving duration of slowing. The resulting estimates of TOSD and duration of slowing can be found in Table S2.

**Statistical Analysis of Behavior – Stop Task.** In keeping with the recommendations based on Monte Carlo simulations [48], we estimated SSRT as the *n^th^* percentile of the No Signal RT distribution minus the signal delay, where *n* is the proportion of errors observed at each signal delay. This estimate was averaged across the signal delays yielding 15% to 85% accuracy for each subject to generate the recommended dependent measure for Stop Tasks with fixed interstimulus intervals (SSRT_AV_). Data from the Stop Task confirmed assumptions of the race model: RTs were faster on Signal trials than on No Signal trials (t(145) = 11.31, p<.0005) and accuracy was a monotonically decreasing function of interstimulus interval (100 vs 150: t(145) = 13.52, p<.0005; 150 vs 250: t(145) = 17.20, p<.0005; 250 vs 300: t(145) = 7.14, p<.0005). These and other behavioral indices of performance across tasks are also reported in Table S3.

## Supporting Information

Figure S1 **Stimuli used in the three experiments.** (A) Experiment 1 included null trials consisting only of a fixation ring, constituting 33% of the total number of trials. Of the remaining trials, 75% were No-Signal trials – i.e., 2AFC trials in which either a left-pointing or right-pointing arrow was presented. 25% were Signal trials, in which a white box followed the onset of the 2AFC stimulus. (B) Experiments 2 & 3 used this slightly different set of stimuli, in which the arrows were replaced with left- or right-pointing triangles, and the number of illuminated pixels was matched between the triangles and squares.
(TIF)

Figure S2 **rVLPFC ROIs were used in the univariate and multivariate fMRI analyses.** Subregions of the rVLPFC include Brodmann Areas 44 (blue), 45 (red), and 47 (green).
(TIF)

Figure S3 **MVPA methods.** (A). For classifying subjects, neural networks received inputs consisting of 1 unit per voxel in a given ROI, where the activity of those units corresponds to the z-transformed and trimmed parameter estimates from the unsmoothed BOLD data. This input layer projects to a hidden “Scalar Val” layer, which transforms each input unit's activity into a distributed pattern across 30 dedicated units. Finally, this hidden layer is fully connected with an output layer consisting of 18 units, one corresponding directly to each of our subjects. (B). For classifying trial types, we used the same architecture as in A except that only 2 output units were used, corresponding directly to each of the trial type contrasts. In addition, separate networks were trained for each subject.
(TIF)

Figure S4 **Tasks can be discriminated in all ROIs, including V1.** Although tasks were best classified on the basis of the Signal > Null contrast (white bars), this is unlikely to reflect stopping-specific processes, since activity patterns in V1 allowed the best classification on this contrast. Indeed, V1 showed the best classification of tasks across all ROIs, when averaging across contrasts. Because our tasks were collected in separate runs, this good classification performance is likely to reflect run-specific variance, rather than task-specific variance. This conclusion is further supported by above-chance discrimination of tasks on the basis of nuisance trials, during which both stimuli and responses were precisely matched across tasks/runs.
(TIF)

Figure S5 **Scalp topographies of the P3.** The typical pattern of “P3 anteriorization” in tasks that demand stopping was reversed in our tasks, such that Double Go_Signal_ trials elicited a larger P3 than the Stop_Signal_ trials at the site where the Stop P3 is typically maximal (A). In contrast, the opposite was true of more posterior electrodes (B & C), indicating that anteriorization effects cannot not be taken to index explicit motoric stopping demands.
(TIF)

Figure S6 **The group-average scalp distribution of ERPs elicited by Stop_Signal_ and Double Go_Signal_ trials were markedly similar, consistent with the strong relationship of these ERPs at the level of individual differences.** In particular, the anteriorization of the P3 ERP elicited by Double Go_Signal_ trials, relative to that elicited by Stop_Signal_ trials, is visible in the highlighted portion of each figure. Each contour represents a change of .79 µV; red is positive.
(TIF)

Figure S7 **Schematic illustration of the process models of our tasks.** (A). The race model is used to analyze behavior in the Stop task, such that the amount of warning necessary to stop (Stop Signal Reaction Time, or SSRT) can be extracted as the *n* ^th^ percentile of the Stop_No-Signal_ distribution, where *n* corresponds to the percent of unsuccessfully stopped responses at a particular signal delay. (B) A conceptually similar model is used to analyze behavior in the Double Go Task, but allows the extraction of *two* underlying parameters. The duration of slowing can be estimated as the difference between slowed 1^st^ responses on Double Go_Signal_trials and responses of the same percent rank on Double Go_No-Signal_ trials. The time of signal detection can be estimated as the amount of time that must elapse following a signal before responses are slowed. (C) The process model of the Double Go Task predicts that slowing should be larger when signals are presented earlier; this prediction was confirmed.
(TIF)

Table S1 **Differences between Experiments 1–3.**
(DOCX)

Table S2 **Mixture model estimates.**
(DOCX)

Table S3 **Descriptive statistics for model-based analyses across Experiments.**
(DOCX)

Table S4 **Statistical results from all primary analyses both with and without those trials designated by the mixture model as “slowed” in the Go task.**
(DOCX)

Text S1 **Additional methods, results, and discussion.**
(DOC)
