# Supplementary figures and images for: Cognitive Control Reflects Context Monitoring, Not Motoric Stopping, in Response Inhibition
Source: PLoS One. 2012 Feb 27;7(2):e31546. doi: 10.1371/journal.pone.0031546 (PMC3288048; doi:10.1371/journal.pone.0031546)

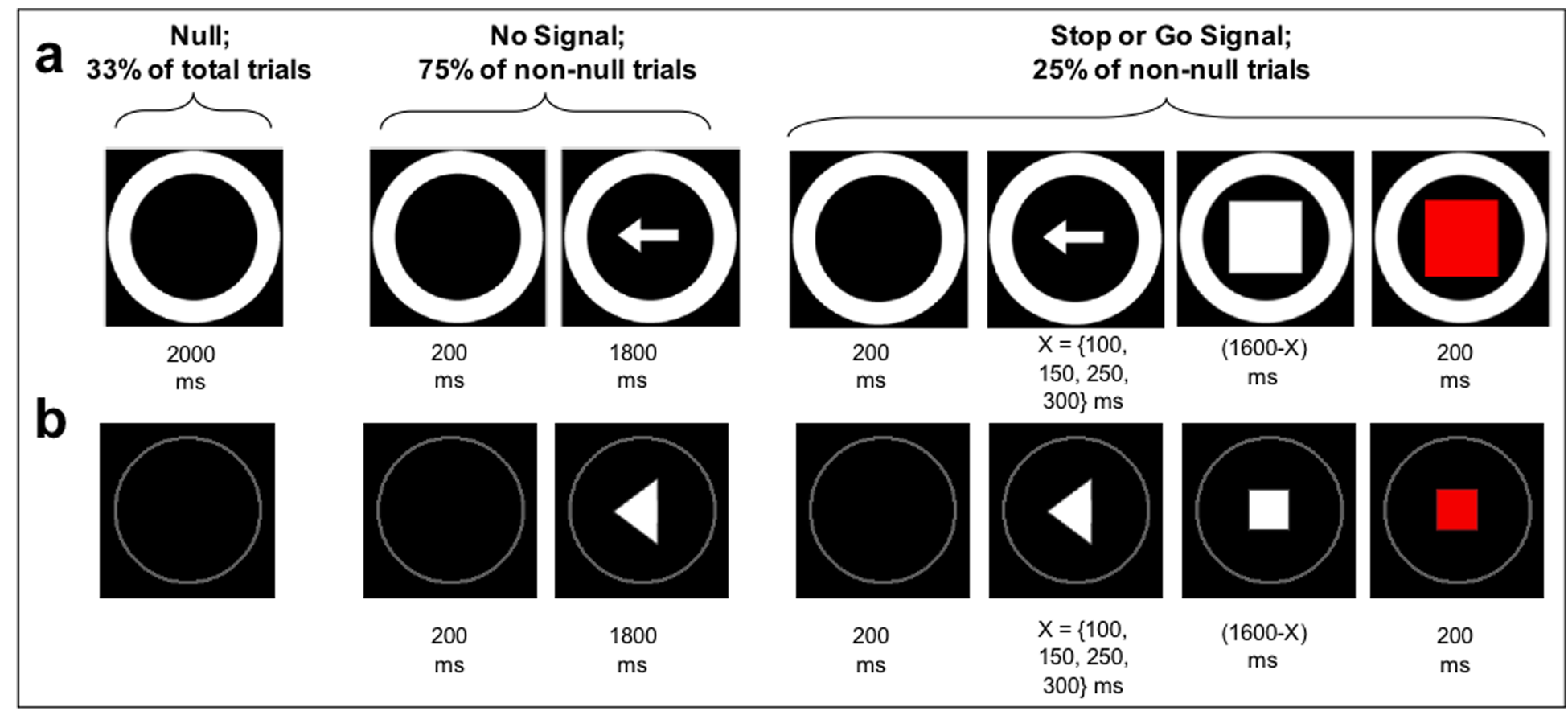

Supplement: Figure S1 — Stimuli used in the three experiments. (A) Experiment 1 included null trials consisting only of a fixation ring, constituting 33% of the total number of trials. Of the remaining trials, 75% were No-Signal trials – i.e., 2AFC trials in which either a left-pointing or right-pointing arrow was presented. 25% were Signal trials, in which a white box followed the onset of the 2AFC stimulus. (B) Experiments 2 & 3 used this slightly different set of stimuli, in which the arrows were replaced with left- or right-pointing triangles, and the number of illuminated pixels was matched between the triangles and squares. (TIF) [file pone.0031546.s001.tif]

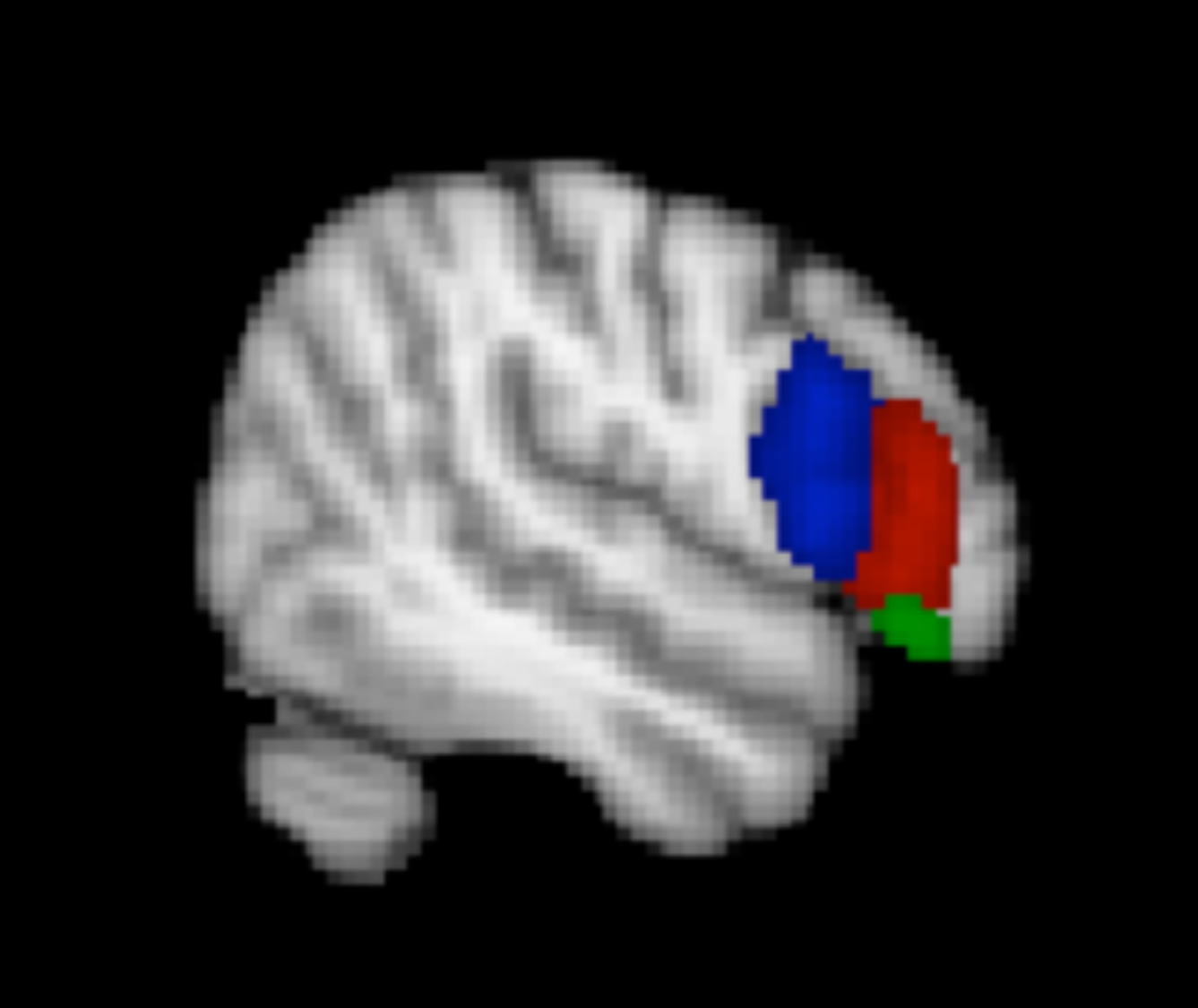

Supplement: Figure S2 — rVLPFC ROIs were used in the univariate and multivariate fMRI analyses. Subregions of the rVLPFC include Brodmann Areas 44 (blue), 45 (red), and 47 (green). (TIF) [file pone.0031546.s002.tif]

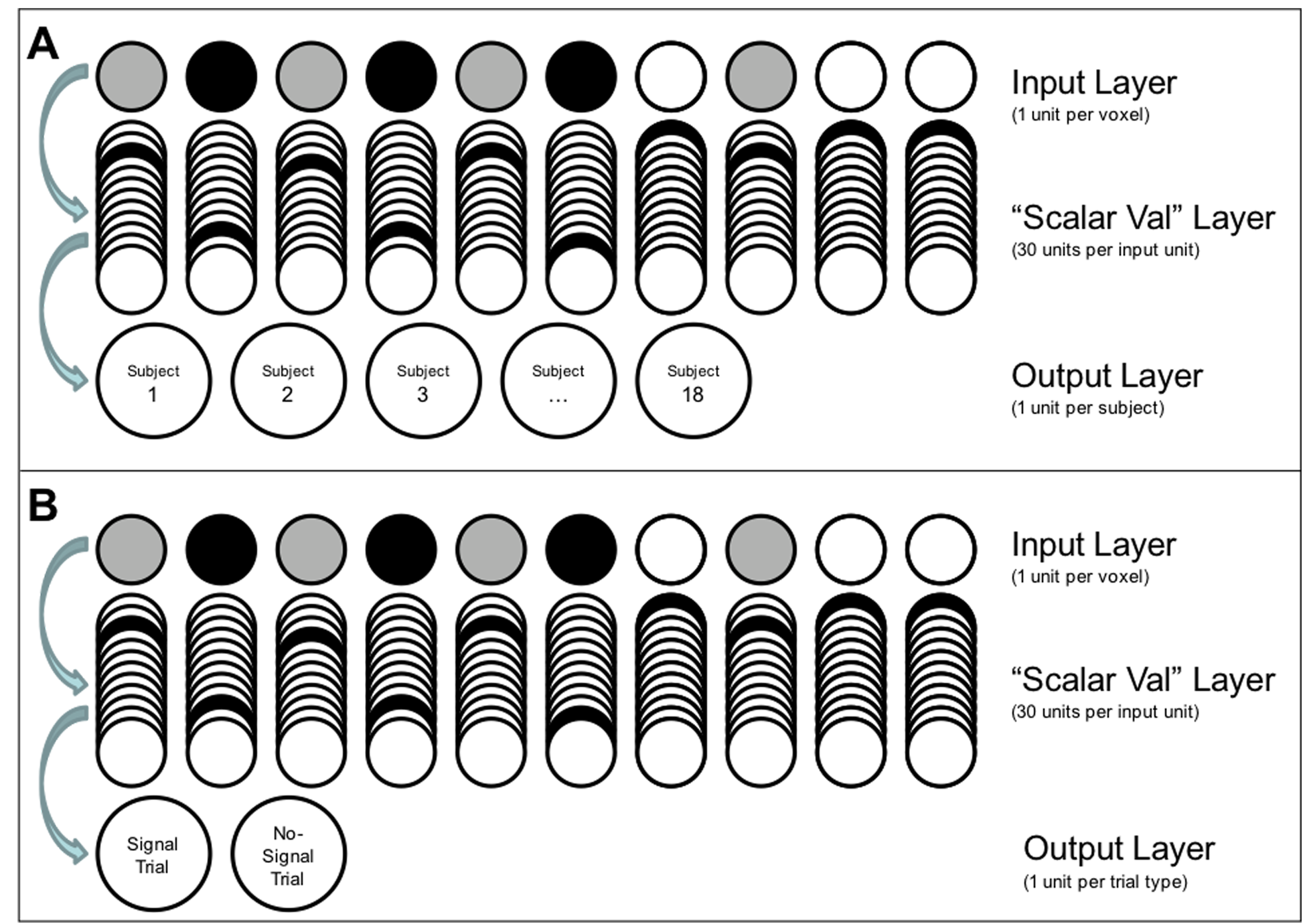

Supplement: Figure S3 — MVPA methods. (A). For classifying subjects, neural networks received inputs consisting of 1 unit per voxel in a given ROI, where the activity of those units corresponds to the z-transformed and trimmed parameter estimates from the unsmoothed BOLD data. This input layer projects to a hidden “Scalar Val” layer, which transforms each input unit's activity into a distributed pattern across 30 dedicated units. Finally, this hidden layer is fully connected with an output layer consisting of 18 units, one corresponding directly to each of our subjects. (B). For classifying trial types, we used the same architecture as in A except that only 2 output units were used, corresponding directly to each of the trial type contrasts. In addition, separate networks were trained for each subject. (TIF) [file pone.0031546.s003.tif]

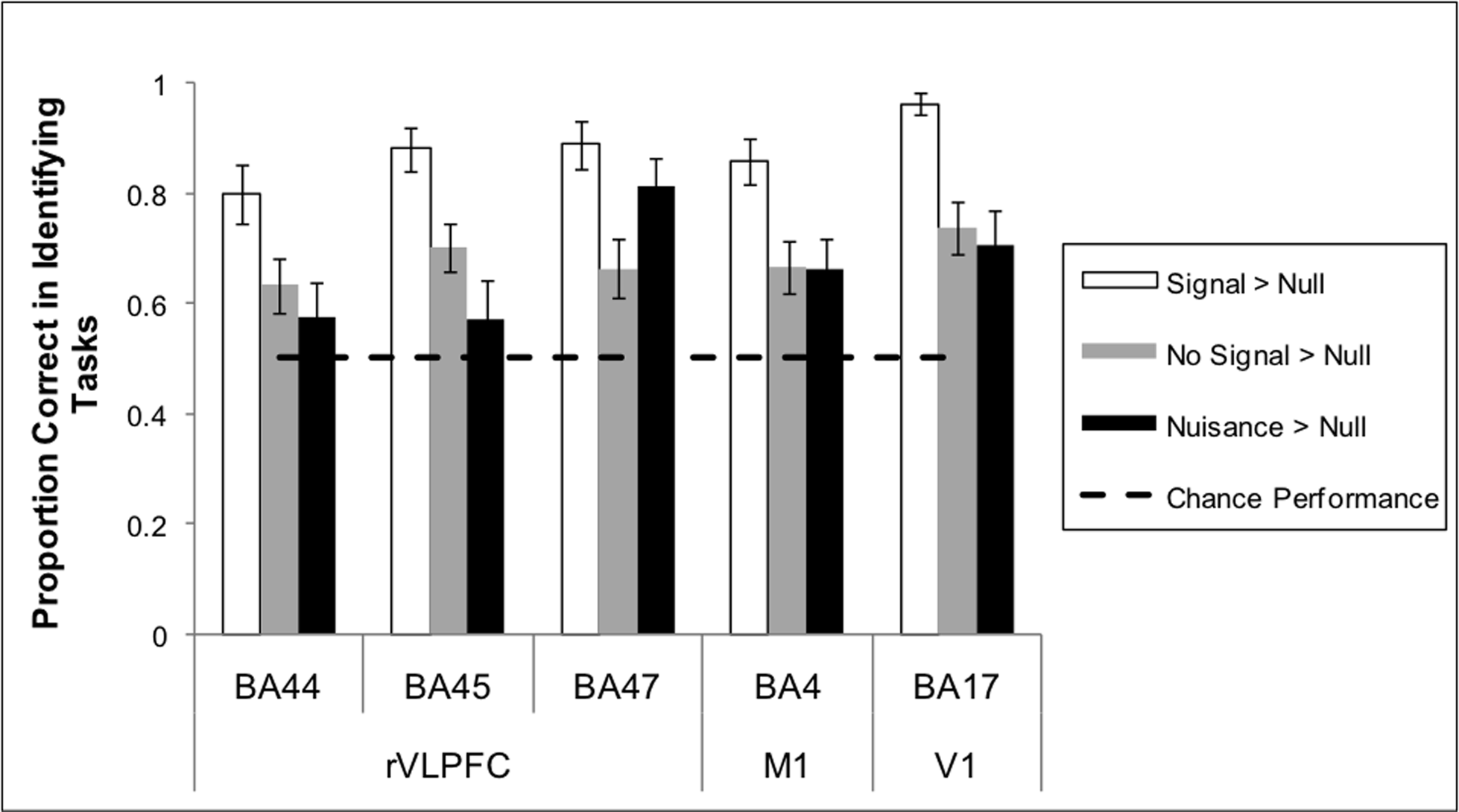

Supplement: Figure S4 — Tasks can be discriminated in all ROIs, including V1. Although tasks were best classified on the basis of the Signal > Null contrast (white bars), this is unlikely to reflect stopping-specific processes, since activity patterns in V1 allowed the best classification on this contrast. Indeed, V1 showed the best classification of tasks across all ROIs, when averaging across contrasts. Because our tasks were collected in separate runs, this good classification performance is likely to reflect run-specific variance, rather than task-specific variance. This conclusion is further supported by above-chance discrimination of tasks on the basis of nuisance trials, during which both stimuli and responses were precisely matched across tasks/runs. (TIF) [file pone.0031546.s004.tif]

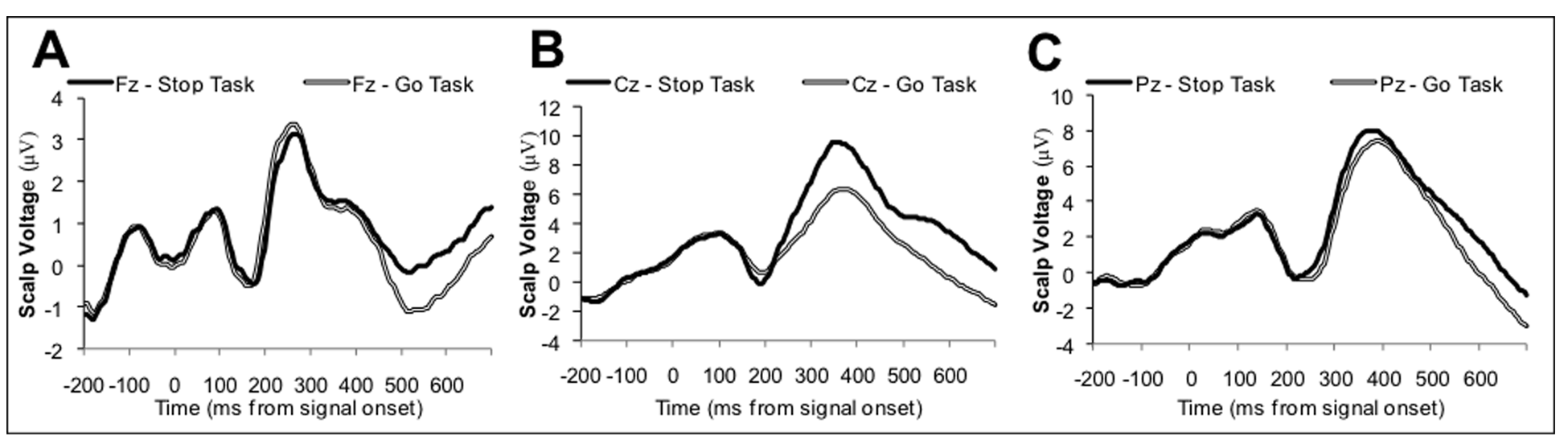

Supplement: Figure S5 — Scalp topographies of the P3. The typical pattern of “P3 anteriorization” in tasks that demand stopping was reversed in our tasks, such that Double GoSignal trials elicited a larger P3 than the StopSignal trials at the site where the Stop P3 is typically maximal (A). In contrast, the opposite was true of more posterior electrodes (B & C), indicating that anteriorization effects cannot not be taken to index explicit motoric stopping demands. (TIF) [file pone.0031546.s005.tif]

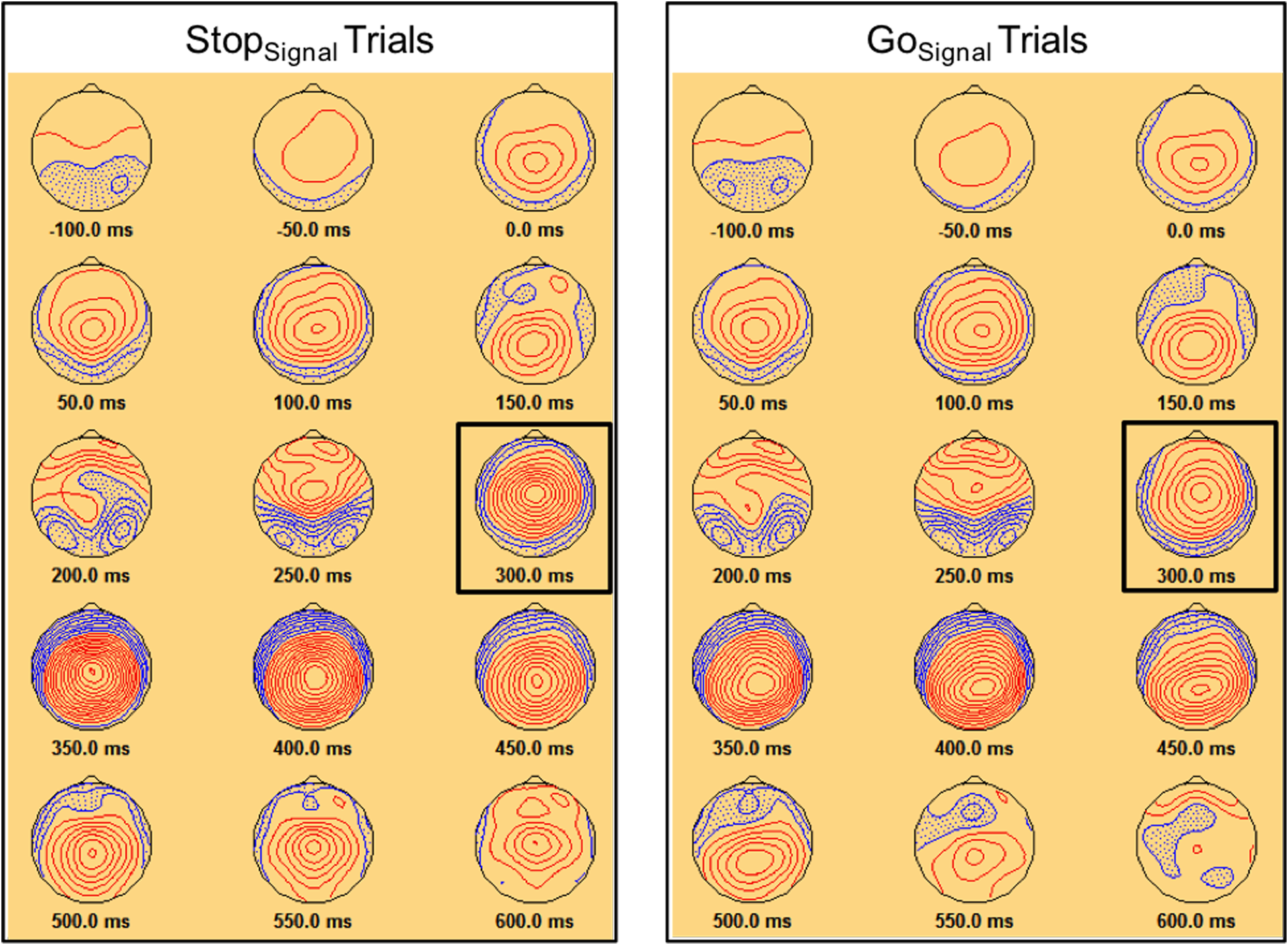

Supplement: Figure S6 — The group-average scalp distribution of ERPs elicited by StopSignal and Double GoSignal trials were markedly similar, consistent with the strong relationship of these ERPs at the level of individual differences. In particular, the anteriorization of the P3 ERP elicited by Double GoSignal trials, relative to that elicited by StopSignal trials, is visible in the highlighted portion of each figure. Each contour represents a change of .79 µV; red is positive. (TIF) [file pone.0031546.s006.tif]

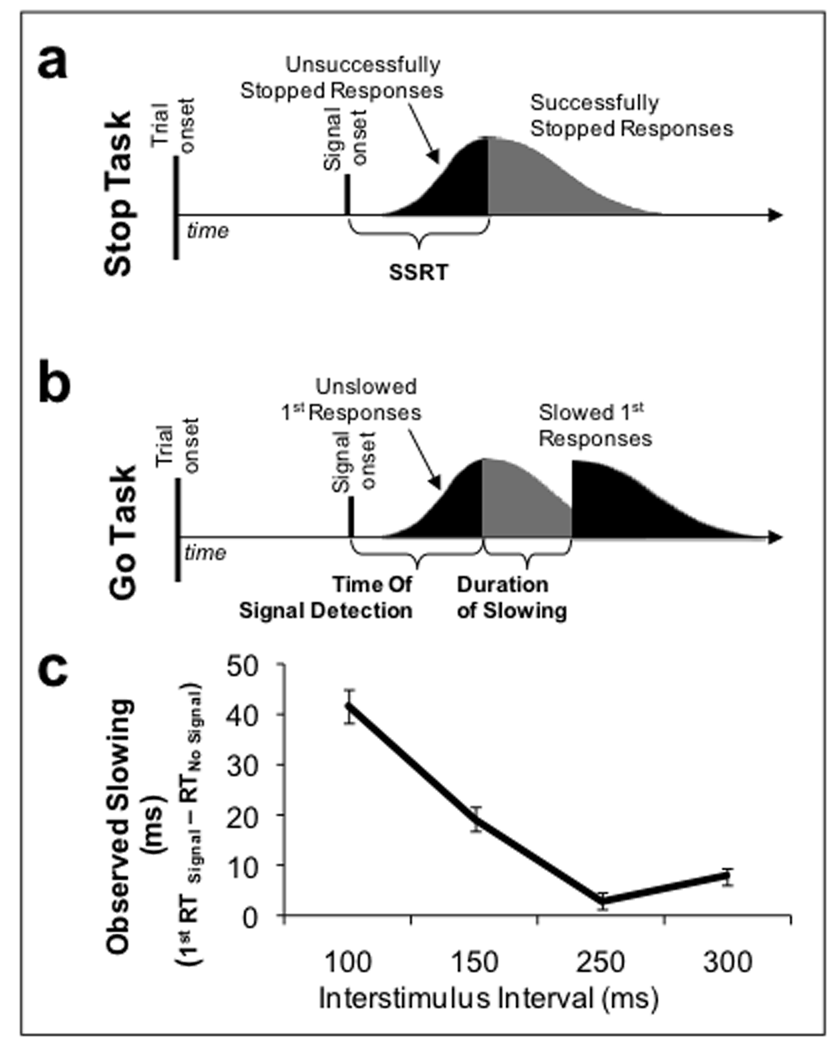

Supplement: Figure S7 — Schematic illustration of the process models of our tasks. (A). The race model is used to analyze behavior in the Stop task, such that the amount of warning necessary to stop (Stop Signal Reaction Time, or SSRT) can be extracted as the n th percentile of the StopNo-Signal distribution, where n corresponds to the percent of unsuccessfully stopped responses at a particular signal delay. (B) A conceptually similar model is used to analyze behavior in the Double Go Task, but allows the extraction of two underlying parameters. The duration of slowing can be estimated as the difference between slowed 1st responses on Double GoSignaltrials and responses of the same percent rank on Double GoNo-Signal trials. The time of signal detection can be estimated as the amount of time that must elapse following a signal before responses are slowed. (C) The process model of the Double Go Task predicts that slowing should be larger when signals are presented earlier; this prediction was confirmed. (TIF) [file pone.0031546.s007.tif]
