# Supplementary material for: Cognitive Control Reflects Context Monitoring, Not Motoric Stopping, in Response Inhibition
Source: PLoS One. 2012 Feb 27;7(2):e31546. doi: 10.1371/journal.pone.0031546 (PMC3288048; doi:10.1371/journal.pone.0031546)
Supplement: Table S1 — Differences between Experiments 1–3. (DOCX) [file pone.0031546.s008.docx]

**Supporting Table 1**

|  | **Experiment** | | |
| --- | --- | --- | --- |
|  | **Exp. 1** | **Exp. 2** | **Exp. 3** |
| # of trials (trials per block) | 400 (100) | 970 (46.19) | 486 (60.75) |
| Luminance-matched signal and no signal trials | No | Yes | Yes |
| Feedback | Sham for Double Go Task, veridical for Stop task | Veridical for both | Veridical for both |
