# Supplementary material for: Cognitive Control Reflects Context Monitoring, Not Motoric Stopping, in Response Inhibition
Source: PLoS One. 2012 Feb 27;7(2):e31546. doi: 10.1371/journal.pone.0031546 (PMC3288048; doi:10.1371/journal.pone.0031546)
Supplement: Table S2 — Mixture model estimates. (DOCX) [file pone.0031546.s009.docx]

**Supporting Table 2.**

| Model | Maximum Likelihood Estimates | | | | BIC |
| --- | --- | --- | --- | --- | --- |
|  | Μ | σ | *k* | ϴ |  |
| Gaussian/Gamma Mixture | -  (fixed to 0) | 22.99 | 4.65 | 16.45 | 39654.22 |
| Gaussian | 15.15 | 33.41 | - | - | 40421.96 |
