# Supplementary material for: Cognitive Control Reflects Context Monitoring, Not Motoric Stopping, in Response Inhibition
Source: PLoS One. 2012 Feb 27;7(2):e31546. doi: 10.1371/journal.pone.0031546 (PMC3288048; doi:10.1371/journal.pone.0031546)
Supplement: Table S3 — Descriptive statistics for model-based analyses across Experiments. (DOCX) [file pone.0031546.s010.docx]

**Supporting Table 3**

| Measure | Exp. 1 | Exp. 2 | Exp. 3 |
| --- | --- | --- | --- |
| SSRT_AV_(sd) | 216ms (33) | 206ms (45) | 232ms (40) |
| TOSD(sd) | 246ms (60) | 203ms (66) | 200ms (41) |
| Duration of Slowing (sd) | 60ms (25) | 72ms (21) | 56ms (14) |
| Pearson R:  TOSD vs SSRT_AV_ | .38 (p<.0005) | .46 (p=.002) | .61 (p=.006) |
| Pearson R:  Slowing Duration vs SSRT_AV_ | -.18 (p>.1) | -.246 (p>.1) | .43 (p=.1)* |
| Double Go TaskRT_Signal_(sd) | 387ms (45) | 363ms (54) | 361ms (41) |
| Double Go TaskRT_No Signal_(sd) | 371ms (35) | 343ms (36) | 349ms (34) |
| Stop TaskRT_Signal_(sd) | 379ms (36) | 353ms (40) | 366ms (40) |
| Stop Task RT_No Signal_(sd) | 426ms (74) | 381ms (50) | 383ms (59) |

* - a single outlier contributed strongly to this positive trend. Exclusion of that subject led to a highly non-significant correlation (p > .49) while not substantially affecting the TOSD vs SSRT_av_correlation (which actually became more significant, with the p value reduced to .001)
