# Supplementary material for: Cognitive Control Reflects Context Monitoring, Not Motoric Stopping, in Response Inhibition
Source: PLoS One. 2012 Feb 27;7(2):e31546. doi: 10.1371/journal.pone.0031546 (PMC3288048; doi:10.1371/journal.pone.0031546)
Supplement: Table S4 — Statistical results from all primary analyses both with and without those trials designated by the mixture model as “slowed” in the Go task. (DOCX) [file pone.0031546.s011.docx]

**Supporting Table 4**

| **Critical Tests  Involving Double Go_Signal_ trials** | **Analysis of All Correct Trials (As described in main text)** | **Re-analysis after Excluding Trials Categorized as “Slowed”** |
| --- | --- | --- |
| Univariate fMRI; Transient Recruitment across ROIs:  Contrasts of Percent Signal Change  (Double Go Task > Stop Task) | STN: t(17)=5.49, p<.0001  BA 44: t(17)=5.08, p<.0001  BA 45: t(17)=2.83, p=.012  BA 47: t(17)=2.5, p=.023  Interaction with TPJ: F(1,17)=31.57, p<.0001 | STN: t(17)=4.18, p=.001  BA 44: t(17)=4.11, p=.001  BA 45: t(17)=2.75, p=.014  BA 47: t(17)=3.38, p=.004  Interaction with TPJ:  F(1,17)=14.82, p=.001 |
| Univariate fMRI:  Mean Percent Signal Change for Sustained rVLPFC Activity Within Double Go Task  (and t-statistics) | BA 44: M= .139  t(17)=2.76, p=.01  BA 45: M=.284  t(17)=4.51, p<.001  BA 47: M=.211  t(17)=3.37, p<.005 | BA 44: M=.144  t(17)=2.91, p=.01  BA 45: M= .319  t(17)=4.95, p<.001  BA 47: M=.234 t(17)=3.66, p=.002 |
| fMRI MVPA; Classification of Individual Subjects:  Contrasts of Performance on Signal vs. No Signal trials | BA44: t(9)=13.5, p<.0001;  BA45: t(9)=11.39, p<.0001;  BA47: t(9)=12.35, p<.001  Interaction with M1:  F(1,9)=85.12, p<.0001 | BA44: t(9)=11.84, p<.0001;  BA45: t(9)=14.10, p<.0001;  BA47: t(9)=8.29, p<.001  Interaction with M1:  F(1,9)=200.132, p<.0001 |
| fMRI MVPA; Classification of Trial Types:  Interactions of D-Prime across ROIs | Interaction of rVLPFC’s BA’s (44, 45 and 47) with M1:  F(1,17)=13.14, p<.005 | Interaction of rVLPFC’s BA’s (44, 45 and 47) with M1:  F(1,17)=9.17, p<.01 |
| ERPs: Stop P3 Amplitude Comparison Across Tasks | t(35)=2.92, p<.03 | t(35)=2.19 p<.04 |
| ERPs: Correlation of Scalp Voltages Across Tasks Following Signal Onset | Pearson R:  Median: .815 Range: .429-.890 | Pearson R:  Median: .805 Range: .462-.871 |
| ERPs: Change in Correlation After Signal Onset – Interaction with Montage | Interaction of Frontal vs. Occipital Electrodes:  F(1,98)=12.59, p=.001 | Interaction of Frontal vs. Occipital Electrodes:  F(1,98)=46.79, p<.0005 |
| Pupillometry: Comparison of average pupil diameter across trial types | Stop_Signal_<Double Go_Signal_  t(85)=13.67, p<.001 | Stop_Signal_<Double Go_Signal_  t(85)=13.02, p<.001 |
