# Supplementary material for: Cognitive Control Reflects Context Monitoring, Not Motoric Stopping, in Response Inhibition
Source: PLoS One. 2012 Feb 27;7(2):e31546. doi: 10.1371/journal.pone.0031546 (PMC3288048; doi:10.1371/journal.pone.0031546)
Supplement: Text S1 — Additional methods, results, and discussion. (DOC) [file pone.0031546.s012.doc]

**Supporting Text for**

Cognitive control reflects context monitoring, not motoric stopping, in response inhibition

Christopher H. Chatham^1*^, Eric D. Claus^2^, Albert Kim^3^, Tim Curran^3^, Marie T. Banich^4^, Yuko Munakata^3^

^1^ Department of Cognitive, Linguistic and Psychological Sciences, Brown University, Providence, Rhode Island, USA

^2^ The Mind Research Network, Albuquerque, New Mexico, USA

^3^ Department of Psychology and Neuroscience, University of Colorado, Boulder, Boulder Colorado, USA

^4^ Institute of Cognitive Science, University of Colorado, Boulder, Boulder Colorado, USA

^*^ Corresponding author: chathach@gmail.com

**This file includes:**

**Supporting Methods and Results**

**Supporting Discussion**

**Supporting References**

**Supporting Methods: Instructions to Subjects**

Instructions to subjects were essentially identical across all experiments. For the Double Go Task in Experiment 1, subjects were told, “In this task you will respond TWICE to arrows that are subsequently covered up by a square. AS QUICKLY AS POSSIBLE, press the z button to left arrows, and the m button to right arrows. If a square appears, press the same button again. Always anticipate the appearance of the square by PREPARING to press the button twice. You MUST respond to squares very quickly - if you're too slow, the square will turn red, and the trial will be marked as incorrect. Keep your fingers on the keys throughout all the tasks. If you have any questions please ask them now.” The instructions for the Double Go Task differed in Experiment 2 in that subject were told to expect frequent “blink breaks,” but should attempt to blink as little as possible throughout the rest of the task. The instructions for the Double Go Task also differed in Experiment 3, in that a MR-compatible button box was used for responding; thus “z button” was replaced with “leftmost button” and “m button” was replaced with “rightmost button” in the instructions. Instructions for Experiment 2 and 3 were otherwise identical to those in Experiment 1. The stop task instructions were as follows in Experiment 1, with changes throughout subsequent experiments that were analogous to those just described for the Double Go Task: “Now you will perform the opposite task. Your task is to respond to the arrows UNLESS the square appears, but you should not wait for the square. We are interested both in how fast you can respond and how well you can stop - both are equally important. Keep your fingers on the buttons so that you can respond to the arrows as soon as they appear. If you see the square, try to STOP yourself from responding. This will sometimes be physically impossible, but try your best. If you fail to stop, the square will turn red. If you have any questions please ask them now.”

We note that while subjects were not told to prepare to stop on all trials within the Stop task (consistent with the standard instructions for that task), subjects *were* told to prepare to respond twice on all trials within the Double Go task. This particular procedure was adopted based on results from extensive piloting of the Double Go task, which indicated that subjects were unlikely to proactively prepare their responses without such instructions (as reflected in unacceptably long RTs on Signal trials, and a large number of omission errors). This stands in stark contrast to results from the Stop task, which indicate that subjects engage proactive control even in the absence of instructions to do so [1-5]. This difference in instructions could thus be reasonably expected to bring the strategies of the two tasks into alignment. Nonetheless, the role of instructions and strategic responding are important directions for future work, given that departures from standard instructions for the Stop task (although such departures did not occur here) are known to influence performance [6].

**Supporting Methods: Behavioral Task Design**

As mentioned in the main text, a fixed task order was adopted for four reasons. First, had tasks been administered in the opposite order, subjects might have associated the infrequent stimulus with the act of stopping in the Stop Task and then potentially recruited stopping processes during the Double Go Task to overcome this prepotent association. Second, the current task order may maximize our ability to detect effortful response inhibition processes, because the act of responding should be more prepotent following the Double Go Task. Third, previous work using a counterbalanced design demonstrated that the univariate hemodynamics of the rVLPFC are similar during stopping and monitoring tasks [7]; because we observed this similarity as well as similarities in multivariate hemodynamics, electrophysiology, pupillometry, and behavior, use of a fixed task order is unlikely to have influenced the results reported here. Fourth, although not as standard in purely experimental work, the use of fixed task orders is a central tenet of individual differences studies [8-10], because counterbalancing can introduce substantial noise into individual difference correlations. Thus the use of a fixed task order allows us to substantially expand upon previous work by providing increased sensitivity to individual differences influencing both stopping and monitoring processes.

Concerns about the impact of task order on our effects are largely addressed by a very recent study which has used intermixed or counterbalanced designs to examine the similarity between a Double Go or Single Go task and the Stop task. Owing to their intermixed designs, these studies are clearly not subject to order effects, and yet reveal results that are conceptually identical to our own [11-12]. In particular, the latter of these studies utilizes the same Double Go task we use here, and finds increased activation to Double Go_Signal_ trials than to Stop_Signal_ trials, which is entirely consistent with our group-level fMRI results. Thus it seems that any order effects would not change our conceptual conclusions or bear on these group analyses of our manuscript. Pupillometry and event-related potentials have not been previously investigated on Double Go trials intermixed with Stop Signal trials; our experiments are the first to use these methods within this task. Thus any arguments about order effects on pupillometry or event-related potentials are necessarily speculative. However, one might expect intermixed designs to also yield: 1) an increased "Stop P3" on Double Go Signal trials, to the extent that the associated increased hemodynamic response in rVLPFC is linked to the P3 [13], and in turn, 2) an increased pupillary response on Double Go Signal trials, to the extent that the P3 and pupillary response are both driven by the LC/norepi system [14-15].

Also as mentioned in the main text, there were minor variations across experiments in the precise design of stimuli, inter-trial intervals, and other characteristics; the visual differences are illustrated in Figure S1, and other differences are described in Table S1.

**Supporting Methods: fMRI Acquisition and Regression Model**

Functional data were collected in a two runs of 216 EPI volumes, each consisting of 32 4 mm thick slices (gap=0 mm, field-of-view (FOV)=220 mm, in-plane matrix= 64 x 64, in-plane resolution= 3.44 x 3.44 mm2), angled parallel to the AC-PC line. Prior to the functional runs, high-resolution T1-weighted 3D IR-SPGR full head anatomical images were acquired along the coronal plane (TR=9 ms, TE=2 ms, flip angle=10º, inversion time=500 ms; 220 mm FOV, 256 x 256 matrix, 0.87 mm x 0.87 mm in-plane resolution, 124 slices, 1.7-mm slice thickness). The scanner was equipped with a standard head coil and participant’s heads were secured with moldable pillows to minimize head motion. Stimuli were displayed through fiber-optic goggles and participants responding by pressing one of two buttons on a fiber-optic button box.

Customized square waveforms were generated for each participant and run. Separate waveforms were generated for No Signal trials with left and right pointing arrows, for Signal trials in which the subject had been correct and incorrect, and for task blocks. In addition, we generated several control waveforms to control for nuisance variables; this included parametric waveforms generated for the number of TRs since the last signal trial and for the interstimulus delay with which each signal was presented, as well square waveforms for cues indicating the onset and offset of task blocks. Finally, a waveform was generated specifying the onset and duration of task blocks. These waveforms were convolved with a double gamma hemodynamic response function (HRF). For each participant, we used FILM (FMRIB’s Improved Linear Model) to estimate the hemodynamic parameters for the different explanatory variables (EVs; e.g. one for each of the separate waveforms) and generate statistical contrast maps of interest (e.g. a contrast between Signal and No Signal trials), with separate models fit to each task. As with the ERP and pupillometry analyses, this procedure ensures the task-evoked hemodynamic response is calculated relative to within-task baselines, thereby controlling for non-specific task differences such as scanner drift or fatigue.

**Supporting Results: univariate fMRI analyses**

In addition to the contrasts reported in the main text, we also contrasted Double Go_No-Signal_ and Stop_No-Signal_ directly, to confirm that any differences between those trial types would not contaminate the contrast of (Double Go_Signal_ -Double Go_No-Signal_) vs. (Stop_Signal_ - Stop_No-Signal_). No significant differences were observed between Double Go_No-Signal_ and Stop_No-Signal_ trials.

As mentioned in the methods of the main text, we defined our temporo-parietal junction and subthalamic nucleus ROIs on the basis of prior work. Our rVLPFC ROIs are illustrated in Figure S2 (with BA44 in blue, BA45 in red, and BA47 in green).

**Supporting Results: fMRI Pattern Classification analyses**

Z-transformed beta-weights from the estimation routines implemented by FSL were provided to the network as inputs, with one input unit per voxel. Each unit in this input layer projected to a distinct set of 30 units designed to encode real valued inputs as a distributed pattern (a ScalarVal layer in Emergent). Thus, unit #1 would code for the value -4.5, unit #30 would code for the value 4.5, and other units interpolate between those values. These patterns were smoothed across adjacent units with a Gaussian smoothing kernel of σ = .105. For example, this smoothing allows unit #1 to code for the value -4.5 but also -4.4 and -4.3, with less activation resulting from values that diverged more from the unit’s preferred value. Next, each of these ScalarVal units were fully connected with random weights to all output units, of which there were 18 for classifying individuals (1 per subject; Figure S3A) or 2 for classifying trial types (Signal trials & No-Signal trials; Figure S3B).

As mentioned in the main text, separate networks were then trained for each ROI (and therefore differed in terms of the number of input units), and for identifying which individuals generated the data vs. what trial type the data was estimated from (and therefore differed in terms of the number of output units) but all other aspects of the network architecture were the same. Specifically, all ScalarVal layers contained 30 times the number of input units for that network, and all network connection weights were adjusted via Hebbian and contrastive Hebbian learning rules, with the same mixtures (k_hebb_ = .01) and learning rates (0.05). The equation for the Hebbian weight change is:

(2)

and for contrastive Hebbian learning:

$\Delta_{err}w_{ij}=(x_{i}^{+}y_{j}^{+})-(x_{i}^{-}y_{j}^{-})$ (3)

which is subject to a soft-weight bounding to keep within the 0 − 1 range:

$\Delta_{sberr}w_{ij}=[\Delta_{err}]+(1-w_{ij})+[\Delta_{err}]-w_{ij}$ (4)

The two terms are then combined additively with a normalized mixing constant *k_hebb_*:

$\Delta w_{ij}=\in[k_{hebb}(\Delta_{hebb})+(1-k_{hebb})(\Delta_{sberr})]$ (5)

For classifying individuals, we trained a set of 40 networks (10 each for BA44, 45, 47 and 4) to activate one of 18 output units corresponding directly to which of our 18 subjects generated the z-transformed beta weight input values from the contrast Signal > Null in the Double Go Task. We trained a separate set of 40 networks to do the same for the contrast No-Signal > Null in the Double Go Task. For classifying trial types, we trained a set of 180 networks (10 for each subject) to classify which trial type the z-transformed beta weight input values came from in the Double Go Task: either Signal>Null or No-Signal>Null, using two output units corresponding directly to these two contrasts. For classifying trial types, a separate set of 180 networks was trained for each ROI (44, 45, 47 and 4). Once all networks had performed correctly on 4 successive epochs of testing, the learning rate was turned to zero and networks were presented with data from the corresponding individuals, ROIs, and contrasts in the Stop task. Networks were scored as performing at chance on any trial where they activated all output units equally, or as performing correctly if the correct output unit was the most active. These performance scores were then averaged to yield the data presented in main text Fig. 4.

These analyses require that networks generalize not only across tasks but also across runs, because the tasks were collected in separate runs. Although we excluded subjects for excessive motion, some motion artifacts likely contribute to the below-perfect generalization that we observed across tasks.

It is also possible to train the same networks to discriminate the tasks given a particular ROI and contrast, but that discrimination could be explained as a function of the noise that is not specific to our tasks, but rather specific to the separate runs in which the tasks were collected. To demonstrate this, we trained separate sets of 10 networks to discriminate tasks based on activation in each ROI for each individual on odd-numbered trials, and tested them on even-numbered trials, for three separate contrasts: Signal>Null, No-Signal>Null, and Nuisance>Null, where nuisance trials were those where subjects saw the word “RELAX” for 2 seconds. We found that discrimination of tasks/runs (since these are confounded in our data) across these 2160 networks (10 runs of each network x 18 subjects x 4 ROIs x 3 contrasts) was reliably above chance even for the contrast Nuisance>Null (with mean accuracy of 65%), indicating that run-specific rather than task-specific variance is contributing to the classifier’s accuracy. A separate batch of networks was trained to distinguish the tasks based on activity in bilateral primary visual cortex (BA17), which proved to be the best ROI for distinguishing the tasks on average (F(1,17)=8.462, p=.01) and on the Signal > Null contrast in particular (F(1,17)=17.42, p=.001; Figure S4). Thus, the tasks/runs can be discriminated based on nuisance trials; furthermore, BA17 is the most successful at discriminating tasks in general and on the critical Signal > Null contrast in particular. These results indicate that unambiguous inferences about multivariate patterns that discriminate stopping and context monitoring processes cannot be made when infrequent Go and Stop trials are collected in separate runs. Intermixing those trial types into the same run is likely to introduce other problems, such as the strategic prioritization of stopping demands[16], which is one reason we did not adopt this design ourselves.

**Supporting Results: ERP Analyses**

As described in the main text, frontal correlations increased more than occipital correlations following the onset of the signal, relative to changes in correlations that otherwise happen at the same time (i.e., during No Signal trials). Main text Fig. 3C depicts the most focused contrast, which is this three-way interaction of Trial Type (Signal vs. No-Signal) x Montage (frontal vs. occipital) x Time (before vs. after signal onset) (F(1,98)=12.59, p=.001). This three-way interaction is significant both with and without baseline correction of the ERPs, indicating that individual differences in baselines are not driving the effect, and that the influence of any non-specific task effect (e.g., fatigue) on these ERPs is minimal.

Also as described in the main text, stimuli that demand stopping typically elicit a positive-going potential (i.e., the Stop P3) that is frontally enhanced relative to the potentials on trials that require response commission[17-22]. This “anteriorization” effect is so robust across response inhibition paradigms, including the Stop and Go/NoGo Tasks, that some have argued such anteriorization directly indexes response suppression[23]. Thus, one strong prediction of stopping accounts is that the distribution of the P3 elicited by Stop_Signal_ trials should be more anterior than the P3 elicited by Double Go_Signal_ trials. However, and in direct contradiction to this prediction, the P3 elicited on Stop_Signal_ trials was enhanced relative to Double Go_Signal_ trials only at more posterior electrodes, a significantly different pattern than observed over more anterior electrodes (Cz&Pz vs. Fz: F(1,34)=17.81, p<.0005, Figure S5; see also Figure S6). As such, our results actually demonstrate “centralization” of the P3 on Stop_Signal_ trials – suggesting that the anteriorization effect typically observed in the electrophysiology of stopping should not be taken to directly index response inhibition, but rather a more general monitoring process shared across the “signal” trials of our tasks.

The centralized distribution more often characterizes another component typically elicited by stopping tasks: an ERP with a slightly earlier and negative-going central potential known as the N2. This ERP, unlike the Stop P3, has already been previously shown to be functionally non-specific to response inhibition, but is instead thought to reflect the detection of response conflict[24-27]. For this reason, as well as the fact that source localization demonstrates the N2 has a source in the anterior cingulate – not the rVLPFC [28-29] – the N2 is not of primary interest here. Nonetheless, we note that the N2 was marginally enhanced in the Stop task at central (Cz) electrodes (F(1,34)=4.6, p<.05). In this case, the enhanced N2 may reflect the additional response conflict in the Stop task (where planned responses may have to be unpredictably cancelled) relative to the Double Go Task (where planned responses are always committed).

As described in the main text, the scalp distributions of the two tasks’ ERPs were strikingly similar in terms of individual differences (main text Fig. 5). This similarity is clearly visible even in the group average, such that even relatively subtle features of the group average ERPs are matched across both time and space (Figure S6). The “centralization” of the P3 during the Stop task, relative to the Double Go Task, is also visible in these group averages – specifically at 300ms after signal onset (highlighted regions of Figure S6).

**Supporting Methods: Behavioral Analysis (identical across all experiments)**

*Double Go Task.* Because response slowing was observed in the Double Go Task, this slowing could confound obvious measures of the efficiency of context-monitoring. For example, reaction times to the infrequent stimulus might be used as a proxy measure of context-monitoring, such that larger reaction times are interpreted to reflect less efficient detection of the signal. However, these reaction times could in fact be large for a subject who very efficiently detects the signal but is also unusually slowed by it[[1]](#footnote-2). As mentioned in the main text, we adopted a model-based approach to confront this confound. This model is first introduced conceptually with respect to its core predictions; the underlying mathematics are described next; analyses of the resulting parameter estimates and verifications of the model’s core predictions are described last.

Conceptually the model is analogous to the race model used to analyze data from the Stop task (Fig. S7A&B). According to these models, responses cannot be affected until the signal has been detected. Thus, the time of signal detection (TOSD) in the Double Go Task can be understood as the amount of time that must elapse once a signal is presented until responses are affected by slowing. The first parameter to be estimated, therefore, is whether any given trial is “slowed” or “unslowed”; once this has been determined for each trial, we then estimate for each subject the time that must elapse after signal presentation until responses are categorized as “slowed.” The first core prediction of the model is that this measure should positively correlate with SSRT because both measures contain variance related to the efficiency of signal detection.

However, these measures may also share variance related to the efficiency of a putative inhibitory or motoric stopping process – a process that gives rise to slowing in the Double Go Task, and to stopping in the Stop task. The efficiency of this putative inhibitory process can be independently estimated in the Double Go Task in terms of the amount of slowing experienced by subjects – informally, we are asking “when subjects are slowed, how slowed are they?” This duration of slowing can be estimated as the difference between Double Go_Signal_ trials categorized as “slowed” and corresponding reaction times on Double Go_No-Signal_ trials. We hypothesize that subjects who are more slowed by the signal will not tend to show higher SSRT (i.e., a positive correlation), because we predict that SSRT primarily reflects context-monitoring processes. It is possible that subjects that who are more slowed by the signal will in fact have a stronger inhibitory process (i.e., STN-mediated inhibition is more difficult to overcome), and therefore the duration of slowing may negatively correlate with SSRT.

The model’s third core prediction is that the positive correlation between TOSD and SSRT should remain when controlling for DoS, because both TOSD and SSRT primarily measure context-monitoring, and do not substantially measure any putative inhibitory processes. Verifying all three core predictions requires estimating the full model, but the model’s basic assumptions can be preliminarily validated using a more straightforward prediction. Responses should be less slowed in the Double Go Task when signals are presented late (because the signals will not be detected in time to slow responses). This prediction was confirmed in our data (Figure S7C and main text Fig. 6B).

Having verified this preliminary prediction, we move to estimating the full Double Go Task model. As described above, this requires categorizing each trial as slowed or unslowed. To estimate which trials undergo slowing, we adopted a nonparametric technique based on rank order. In particular, we subtracted from each signal trial RT the observation with corresponding percent rank in the no signal trial RT distribution. To the extent that the two distributions are equivalent, these residuals should be centered on zero. As mentioned in the main text, and consistent with our model, many residuals were centered on zero but there was also a pronounced positive skew to the distribution of these residuals, indicative of slowing for some trials. In contrast to the positive skew typically observed in RTs, this skew was sufficiently strong to be essentially unaffected by logarithmic or square-root transformation.

Our mixture modeling approach decomposed the distribution into two underlying distributions: a Gaussian distribution with a mean of zero (corresponding to unslowed RTs), and a Gamma distribution (corresponding to the slowed RTs). Our choice of the Gamma distribution for slowed RTs was motivated by the fact that the two parameters determining the shape of the Gamma (scale and rate) can generate positively skewed pseudo-normal distributions (which would appear to match the observed distribution of skewed residuals) but can also generate exponential distributions, sometimes used to model queue and waiting times – seemingly a good candidate for the functional form of response slowing. The two free parameters to the Gamma and the one free parameter to the Gaussian yielded a better overall fit than a single Gaussian (Table S2).

Individual RTs were categorized as belonging to the slowed distribution if there was even weak evidence in favor of the RT belonging to that distribution (as quantified by a difference in BIC of ≥ 2.35); otherwise RTs were assigned to the unslowed distribution. We adopted this weak standard of evidence for classification to ensure that any observation that might have undergone slowing would be classified as such. Other standards of evidence lead to similar results as those presented here, but do not as cleanly separate the slowed and unslowed trials (c.f. main text Fig. 6D, where unslowed Double Go_Signal_ trials show a nonsignificant difference from corresponding Double Go_No-Signal_ trials.).

Consistent with the Double Go Task model’s first core prediction, SSRT and TOSD were positively correlated when collapsing across all three experiments (R=.418, p<.0005) and in each experiment individually (Table S3). This positive correlation indicates that SSRT largely reflects the efficiency of context monitoring, as reflected in the time of signal detection.

We also did not observe a positive correlation between SSRT and TOSD, consistent with the Double Go Task model’s second core prediction. In fact, SSRT and slowing duration were negatively correlated when collapsing across all three experiments (R=-.188, p<.05), indicating that those with stronger inhibition (i.e., more slowing) show better performance in the Stop task (i.e., smaller SSRTs). This is also consistent with our model, although this relationship is fairly weak, and thus does not constitute particularly strong support for it.

Finally, we tested the model’s third core prediction by performing a partial correlation of TOSD and SSRT, controlling for the duration of slowing. The robust positive correlation between SSRT and TOSD remained (R=.41, p<.0005), indicating that any variance related to inhibitory processes is not strongly measured by either SSRT or TOSD, relative to the variance in context-monitoring captured by these measures. In other words, the individual differences variance in motoric stopping/slowing processes captured by DoS does not overlap with the individual differences variance that is shared by SSRT and TOSD, indicating that the commonality of TOSD and SSRT does not reflect a common motoric stopping process. This conclusion is concordant with a subsequent re-analysis of our data, presented below, which demonstrates that the overlapping task variance in univariate and multivariate hemodynamics, event-related potentials, and the relative patterns of mental effort do not substantially change when only those Double Go task trials categorized as “unslowed” are analyzed.

**Supporting Methods: Analyses of only *un*slowed Double Go_Signal_ trials**

To further test our hypothesis that the commonalities of the Double Go and Stop tasks do not reflect a common process of motoric stopping or motor plan replacement, we re-analyzed the fMRI, ERP, and pupillometric data including only those trials that were categorized as “unslowed.”

For fMRI, this entailed the respecification of the design matrix for each individual subject. Trials categorized as “slowed” were separately modeled with a new boxcar regressor, and convolved with a double gamma hemodynamic response function (just like our other regressors). These same trials were then omitted from all other regressors (except for the sustained regressor) to avoid issues related to colinearity. This technique allows the hemodynamic response to slowed trials to be separately estimated, and therefore not contaminate the contrasts of transient hemodynamic activity across tasks, nor to contaminate the estimates of percent signal change for the sustained hemodynamic activity across task blocks. This re-analysis revealed similar patterns as we had observed across all Double Go_Signal_ trials: univariate transient hemodynamics were still observed throughout the rVLPFC on unslowed Double Go_Signal_ trials, and these effects were still significantly larger than those observed on Stop_Signal_ trials (Table S4, row 1). This result indicates that the increased hemodynamic response to Double Go_Signal_ trials is not driven by the motoric slowing that is captured by “slowed” trials within the Double Go task.

Likewise, this analysis also replicated our previous finding of sustained activity across all trials in the Double Go task (Table S4, row 2). This finding again indicates that context-monitoring processes, and not motoric stopping or slowing processes, contribute to the sustained activity that is recruited across all trials within the Double Go task.

For the ERP data, this re-analysis entailed re-segmenting the original timeseries of each recording session so that trials categorized as “slowed” could be given their own category, and then excluded from subsequent analysis steps. Otherwise, all ERP analysis procedures were then performed as in the primary analysis, including 40Hz filtering, bad channel replacement, average referencing and polar average reference correction, stimulus locking, baseline correction, montage averaging, and ERP correlations. This re-analysis once again replicated our primary analyses, such that the so-called “Stop P3” was in fact significantly *enhanced* on unslowed Double Go_Signal_ trials relative to Stop_Signal_ trials (Table S4, row 5), indicating that this ERP does not reflect stopping-specific processes. Indeed, all recorded ERPs were still strongly correlated across tasks, indicating that this similarity at the group level was paralleled by similarities in ERPs at the level of individual subjects, even when all of the “slowed” Double Go_Signal_ trials were excluded from analysis (Table S4, row 6). Finally, these correlations were again disproportionately increased over frontal electrodes (relative to occipital ones) in the period following signal onset (Table S4, row 7). By fully replicating our original result when slowed Double Go_Signal_ trials were excluded, this pattern indicates that the increased cross-task similarity in frontal ERPs that is yielded by Signal presentation is not simply driven by the motoric slowing that occurred within the Double Go task.

For the pupillometric data, this re-analysis entailed again resegmenting the original timeseries recording, and omitting the “slowed” Double Go_Signal_ trials from all subsequent analysis steps (which were otherwise identical to those for the primary analysis). Once again, we were able to replicate our original findings after excluding those Double Go_Signal_ trials that had been categorized as slowed, such that pupil diameter was still largest on Double-Go_Signal_ trials than on any other trial type, including Stop_Signal_ trials (Table S4, row 8). In fact, these patterns were slightly enhanced, indicating that the increased mental effort on Signal trials of the Double Go task is not merely driven by any additional effort required for motoric slowing, which itself appears to be negligible.

**Supporting Discussion**

Although the fixed task order used here might be expected to yield greater fatigue in the Stop task, fatigue cannot viably explain at least five prominent features of our results. First, fatigue would predict a reduction in pupil diameter during the Stop task, but instead a larger pupil diameter was observed on Stop_NoSignal_trials than Double Go_NoSignal_ trials. Second, fatigue would predict a global reduction in ERPs in the Stop task, but central and posterior ERPs were enhanced in that task. Third, fatigue would predict global reductions in activation during the Stop task, but areas thought to have domain-general attentional roles showed highly-similar profiles across tasks (e.g., TPJ). Fourth, within-task baselines are used in pupillometry, ERP, and fMRI analysis precisely to control for run-specific effects like fatigue – thus any global differences between tasks (owing to fatigue or more innocuous factors, like scanner drift) are subtractively eliminated in the analyses reported here. Fifth, fatigue would predict that SSRT should be increased in our sample relative to experiments that administer a stop task first, but the range of SSRT observed here was well within normal.

Relatedly, our use of a fixed task order could have yielded some learning effects. For example, subjects clearly had substantial experience in “alerting” to the onset of the Signal by the time they began the Stop task, which could have contributed to the similarities and differences observed between the Double Go and Stop tasks. However, such effects are unlikely to materially influence our primary conclusions. First, rVLPFC responses can be increased after practice with alerting to a particular cue [11], suggesting that the decreased hemodynamic response in the rVLPFC during the Stop task is simply not a result of subjects’ additional experience with alerting to the Signal stimulus. Our pupillometric results are similarly hard to interpret as resulting from learning effects; if such a learning effect exists, it would have to decrease the pupil dilation induced by Signal trials while simultaneously increasing the pupil dilation evoked by No Signal trials. Finally, increased rVLPFC responses have been previously observed to trials that do not require motoric stopping relative to those that do, even when such trials are intermixed in the same run [12]. Nonetheless, it remains a logical possibility that learning effects of this kind do exist; additional studies are necessary (and indeed, in progress) to assess whether they may transfer across tasks with such dissimilar demands on motoric stopping.

We have argued that our ERP and fMRI analyses demonstrating the shared prefrontal substrates of the tasks does not reflect the slowing or stopping process engaged by the Double Go Task. Instead, we suggest that the slowing effect may instead be better understood as an indirect and peripheral consequence of more general attentional processes. For example, cardiac slowing is also observed during tasks involving preparation of a speeded response [30]. Our observation of response slowing and this previous observation of cardiac slowing alike are highly unlikely to reflect a controlled, effortful, or prefrontally-based stopping process. Although subcortical nuclei such as the subthalamic nucleus might be considered more likely to perform these stopping-specific functions, we caution that the subthalamic nucleus is operative in many tasks, not just those requiring an act of stopping. In fact, its activity positively correlates with response force[31], suggesting it may also not have a stopping-specific function. Indeed, others have recently shown a role for the STN in detecting behaviorally relevant stimuli, findings that support a re-evaluation of STN function that is analogous to the current re-evaluation of rVLPFC function[32].

While we have argued that context-monitoring, not stopping, is the cognitively-controlled component to response inhibition tasks, we do not have a position on whether one or both of these processes may be accessible to consciousness. The relationship between consciousness and cognitive control is controversial, with some recent work indicating that rVLPFC can be activated by subthreshold infrequent stimuli that require stopping[33-34]. These results might be taken to contradict accounts that either context-monitoring or stopping is cognitively controlled – at least, under the untested assumption that controlled processes are always consciously accessible. We suggest this assumption warrants empirical test, and is therefore a promising direction for future work.

We also note that cognitive control is not defined by temporal order (if monitoring must occur prior to stopping, this does not imply that monitoring is cognitively controlled) nor solely by prefrontal recruitment (striatum may have cognitive control functions, and the rVLPFC in particular has been previously argued to *not* subserve cognitive control[35]). Instead, assessing cognitive control requires more comprehensive analysis, of the kind we provide here.

**Supporting References**

1. Jahfari S, Stinear C, Claffey M, Verbruggen F, & Aron AR. (2010) Responding with restraint: What are the neurocognitive mechanisms? J Cog Neuro, 22: 1479–1492.
2. Chikazoe J, Jimura K, Hirose S, Yamashita K, Miyashita Y, et al. (2009) Preparation to inhibit a response complements response inhibition during performance of a stop-signal task. J Neurosci 29:15870–15877.
3. Verbruggen F, Logan GD (2009). Proactive adjustments of response strategies in the stop-signal paradigm. JEP:HPP, 35, 835-854.
4. Band GPH, Ridderinkhof KR, Van der Molen MW (2003). Speed-Accuracy Modulation in Case of Conflict: The Roles of Activation and Inhibition. Psychol. Res., 67, 266-279.
5. Liddle EB, Scerif G, Hollis CP, Batty MJ, Groom MJ, et al. (2009) Looking before you leap: a theory of motivated control of action. Cognition 112: 141–158.
6. Leotti LA, Wager TD. (2009) Motivational influences on response inhibition measures. JEP:HPP 36: 430-47.
7. Sharp DJ et al. (2010) Distinct frontal systems for response inhibition, attentional capture, and error processing. Proc Natl Acad Sci U S A 107: 6106-11.
8. Friedman NP, Miyake A, Young SE, Defries JC, Corley RP et al. (2008) Individual differences in executive functions are almost entirely genetic in origin. Journal of Experimental Psychology: General 137: 201-25.
9. Miyake A, Friedman NP, Emerson MJ, Witzki AH, Howerter A, et al (2000) The unity and diversity of executive functions and their contributions to complex "frontal lobe" tasks: A latent variable analysis. Cognitive Psychology 41: 49-100
10. Friedman NP, Miyake A (2004) The relations among inhibition and interference control functions: A latent variable analysis. Journal of Experimental Psychology: General 133: 101-135.
11. Hampshire A, Chamberlain SR, Monti MM, Duncan J, Owen AM (2010) The role of the right inferior frontal gyrus: inhibition and attentional control. Neuroimage 50: 1313-9.
12. Dodds CM, Morein-Zamir S, Robbins TW (2011) Dissociating Inhibition, Attention, and Response Control in the Frontoparietal Network Using Functional Magnetic Resonance Imaging. Cerebral Cortex 21: 1155-1165.
13. Brazdil M, Dobsik M, Mikl M, Hlustik P, Daniel P, et al (2005) Combined event related fMRI and intracerebral ERP study of an auditory oddball task. Neuroimage 26: 285-393.
14. Nieuwenhuis S, De Geus EJ, Aston-Jones G (2011). The anatomical and functional relationship between the P3 and autonomic components of the orienting response. Psychophysiology 48: 162-175.
15. Gilzenrat, MS, Nieuwenhuis S, Jepma M, Cohen JD (2010). Pupil diameter tracks changes in control state predicted by the adaptive gain theory of locus coeruleus function. Cognitive, Affective, and Behavioral Neuroscience 10: 252-69.
16. Morein-Zamir S, Chua R, Franks I, Nagelkerke P & Kingstone A (2007) Predictability influences stopping and response control. JEP:HPP, 33: 149-162.
17. Salisbury DF, Griggs CB, Shenton ME, McCarley RW. (2004) The NoGo P300 'anteriorization' effect and response inhibition. Clinical Neurophysiology 115: 1550-58.
18. Ramautar JR, Kok A, Ridderinkhof K (2006). Effects of stop-signal modality on the N2/P3 complex elicited in the stop-signal paradigm. Biological Psychiatry 72: 96-109.
19. Hämmerer D, Li SC, Müller V, Lindenberger U (2010) An electrophysiological study of response conflict processing across the lifespan: assessing the roles of conflict monitoring, cue utilization, response anticipation, and response suppression. Neuropsychologia 48: 3305-16.
20. Strik WK, Fallgatter AJ, Brandeis D, Pascual-Marqui RD (1998) Three-dimensional tomography of event-related potentials during response inhibition: evidence for phasic frontal lobe activation. Electroencephalogr Clin Neurophysiol. 108: 406-13.
21. Falkenstein M, Hoormann J, Hohnsbein J (1999) ERP components in Go/No Go Tasks and their relation to inhibition. Acta Psychol. 101: 267-91.
22. Fallgatter AJ, Mueller TJ & Strik WK (1999) Age-related changes in the brain electrical correlates of response control. Clin Neurophysiol. 110:833-8.
23. Nieuwenhuis S, Yeung N, van den Wildenberg W, Ridderinkhof KR (2003) Electrophysiological correlates of anterior cingulate function in a go/no-Go Task: effects of response conflict and trial type frequency. Cognit, Affective, and BehavNeurosci 3: 17–26.
24. Pfefferbaum A, Ford JM, Weller BJ & Kopell BS (1985) ERPs to response production and inhibition. Electroenceph & Clin Neurophysiol, 60: 423-434.
25. Azizian A, Freitas AL, Parvaz MA, Squires NK (2006) Beware misleading cues: Perceptual similarity modulates the N2/P3 complex. Psychophysiol 43: 253-260.
26. Donkers FCL, van Boxtel GJM (2004) The N2 in Go/No-Go Tasks reflects conflict monitoring not response inhibition. Brain and Cognit 56: 165-176.
27. Smith JL, Smith EA, Provost AL, Heathcote, A (2010) Sequence effects support the conflict theory of N2 and P3 in the Go/No Go Tak. Int J Psychophysiol. 75: 217-26.
28. Jonkman LM, Sniedt FL, Kemner C (2007) Source localization of the Nogo-N2: a developmental study. Clin Neurophysiol 118: 1069-77.
29. Bekker EM, Kenemans JL, Verbaten MN (2005). Source analysis of the N2 in a cued Go/No Go Task. Brain Res Cogn Brain Res. 22: 221-31.
30. Jennings JR, van der Molen MW, Tanase C (2009) Preparing hearts and minds: cardiac slowing and a cortical inhibitory network. Psychophys. 46: 1170-8.
31. Patil PG, Carmena JM, Nicolelis, MA, Turner DA. (2004) Ensemble recordings of human subcortical neurons as a source of motor control signals for a brain-machine interface. Neurosurgery 55: 27-35.
32. Sauleau P, Eusebio A, Thevathasan W, Yarrow K, Pogosyan A, et al (2009) Involvement of the subthalamic nucleus in engagement with behaviourally relevant stimuli. Eur J Neurosci. 29: 931-42 .
33. van Gaal S, Ridderinkhof KR, Scholte HS, Lamme VA (2010) Unconscious activation of the prefrontal no-go network. J Neurosci 30: 4143-4150.
34. van Gaal S, Ridderinkhof KR, Fahrenfort JJ, Scholte HS, Lamme VA (2008) Frontal cortex mediates unconsciously triggered inhibitory control. J Neurosci 28: 8053-8062.
35. Jiang Y, Saxe R, Kanwisher N. (2004) Functional Magnetic Resonance Imaging Provides New Constraints on Theories of the Psychological Refractory Period. Psychol Sci 15: 390-396.

1. In fact, efficient detection of the signal may lead to more observed slowing precisely because signals will be detected in time to slow responses. This issue confounds model-free estimates of response slowing in tasks with infrequent stimuli that do not demand stopping . [↑](#footnote-ref-2)
